# Regulation of signaling pathways in hair follicle stem cells

**DOI:** 10.1093/burnst/tkac022

**Published:** 2022-07-04

**Authors:** Xiaoxiang Wang, Yinghui Liu, Jia He, Jingru Wang, Xiaodong Chen, Ronghua Yang

**Affiliations:** Department of Burn Surgery, The First People’s Hospital of Foshan, Foshan 528000, China; Guangdong Medical University, Zhanjiang, 524000, China; Department of Dermatology, Dermatology Hospital, Southern Medical University, Guangzhou, 510091, China; Department of Burn Surgery, The First People’s Hospital of Foshan, Foshan 528000, China; Department of Burn Surgery, The First People’s Hospital of Foshan, Foshan 528000, China; Department of Burn Surgery, The First People’s Hospital of Foshan, Foshan 528000, China; Department of Burn Surgery, The First People’s Hospital of Foshan, Foshan 528000, China; Guangdong Medical University, Zhanjiang, 524000, China

**Keywords:** Hair follicle stem cells, Signaling pathways, Proliferation, Differentiation, Regenerative, Repair

## Abstract

Hair follicle stem cells (HFSCs) reside in the bulge region of the outer root sheath of the hair follicle. They are considered slow-cycling cells that are endowed with multilineage differentiation potential and superior proliferative capacity. The normal morphology and periodic growth of HFSCs play a significant role in normal skin functions, wound repair and skin regeneration. The HFSCs involved in these pathophysiological processes are regulated by a series of cell signal transduction pathways, such as lymphoid enhancer factor/T-cell factor, Wnt/β-catenin, transforming growth factor-β/bone morphogenetic protein, Notch and Hedgehog. The mechanisms of the interactions among these signaling pathways and their regulatory effects on HFSCs have been previously studied, but many mechanisms are still unclear. This article reviews the regulation of hair follicles, HFSCs and related signaling pathways, with the aims of summarizing previous research results, revealing the regulatory mechanisms of HFSC proliferation and differentiation and providing important references and new ideas for treating clinical diseases.

## Highlights

- Signaling pathways in HFSCs driving physiological/pathological changes in the HFSC niche have been confirmed.
- In view of HFSC signaling pathways, the mechanisms underlying therapeutic strategies for alopecia and other skin diseases are summarized.
- The crosstalk between signaling pathways in HFSCs and how this determines the fate of HFSCs is also briefly discussed.

## Background

The skin is the largest organ of the body; it covers the surface of the body and contains accessory structural organs such as hair follicles and sebaceous glands. Its basic functions include regulating body temperature, preventing the invasion of pathogens and sensory, secretion and excretion roles. Mammal skin is a tissue with a high regenerative capacity and thousands of hair follicles in the dermis and subcutaneous tissue. The hair follicle is divided into three segments: from top to bottom, the infundibulum, the isthmus and the bulb (extends from the base of the follicle to the insertion of the arrector pili muscle (APM). The growth of the hair follicle is cyclical and can be divided into three phases: anagen (growth), catagen (transition) and telogen (rest) [1]. This periodic activity of hair follicles is controlled by the epithelial–mesenchymal interaction between hair follicle keratinocytes and hair papilla fibroblasts [2,3]. The duration of each phase is different and the duration of different hair cycles is also different; the resting phase is the main phase that controls the hair follicle cycle [4]. Relevant studies have shown that resting-period status does not mean that the hair follicles are in a dormant state, but rather that this is the default state for energy-efficient mammalian fur. The function of the resting phase is to maintain the hair fiber while being able to quickly respond to hair loss [5]. The circulatory process is dominated by hair follicle stem cells (HFSCs) [6], which have characteristically slow periodicity, multi-differentiation potential and strong *in vitro* proliferation ability. HFSCs are adult stem cells and the main stem cell markers that they express are CD34, K15, Gli1, LIM homeobox 2 and SRY-related high mobility group-box gene 9 (SOX9) [7,8]. This group of cells maintain self-renewal and pluripotency throughout their life cycle, leading to the processes of tissue regeneration and wound repair, which are two completely different states *in vivo* and *in vitro* [9,10]. Studies have found that HFSCs are in a static state in the body, but they grow clonally *in vitro*, showing amazing proliferation ability and can differentiate into neuronal cells, glial cells, melanocytes and smooth muscle cells [11–13]. The multi-differentiation potential of HFSCs enables them to differentiate into the epidermis, hair follicles and sebaceous glands, and to participate in the skin wound healing process [14]. Shwartz *et al*. recently found that APMs are crucial for the formation and maintenance of sympathetic innervation of HFSCs [15]. The APM is a piece of smooth muscle between the bulge and the adjacent mesenchyme that is responsible for raising the hair follicles (piloerection) to trap body heat and express emotions. Whether in a steady state or in a cold state, APMs allow sympathetic innervation to activate HFSCs directly through synapse-like connections that deliver the neurotransmitter norepinephrine [16]. HFSCs are involved in the precise regulation of many signaling molecules and signaling pathways in the maintenance and repair of various skin tissues, including lymphoid enhancer factor/T-cell factor (LEF/TCF), Wnt/β-catenin, transforming growth factor-β/bone morphogenetic protein (TGF-β/BMP), Notch, Hedgehog (HH) and phosphoinositide 3 -kinase/protein kinase B (PI3K/AKT) [17]. These signaling pathways interact with each other to form a complex regulatory network, but the abnormal activation of signaling pathways leads to the epigenetic reprogramming of skin cells, leading to the occurrence and development of diseases. For example, a lack of the Wnt signaling pathway may lead to hair follicle substrate and hair formation obstacles [18], and abnormal sonic hedgehog (SHH) expression can lead to the formation of skin cancer.

Skin stem cells are the main resource for the regeneration or repair of skin and skin appendages. Therefore, the role of HFSCs in skin tissue remains an open question. Some studies have suggested that hair follicle cells that are differentiated from hair follicle bulge are HFSCs. One study used ^3^H-thymidine and bromouracil to repeatedly label mice. They found that there were almost no slowly labeled cells in the epidermis, but HFSCs were present in the bulge area [3]. At present, no complete stem cell markers have been identified, so it is still very difficult to localize skin stem cells accurately in most areas of human skin. Likely, because of this limitation, the bulge hypothesis of HFSCs has received wide attention and support. When the skin is injured, in addition to epidermal cells, hair follicle stem cells are also activated to participate in epidermal regeneration and promote wound repair. c-Myc is a downstream gene of β-catenin and its overexpression overmobilizes stem cells to transform them into other cells, eventually leading to stem cell depletion and skin ulcers [19]. Stem cells can also cause a variety of epithelial tumors and skin diseases. It is speculated that epidermal stem cells may be important targets of physical or chemical factors (including carcinogens) and may even damage the epidermis and hair follicle adnexa [20,21]. In view of this possibility, skin stem cells may be used as the first target of skin gene therapy in the future. This article reviews the relationship between HFSCs and major signaling pathways and analyzes the prospects for using HFSCs for treating clinical diseases in the future.

## Review

### LEF/TCF signaling pathway

The LEF/TCF family is a group of transcription factors that play an important role in the growth, development and maintenance of stem cell homeostasis. The LEF/TCF protein family is small, primarily including four types of TCF/LEF proteins in humans, namely, TCF-1, TCF-3, TCF-4 and LEF-1 [19]. LEF/TCF is a sequence-specific DNA-binding transcription factor. The synergistic effect of LEF/TCF and activated β-catenin (Wnt signaling product) acts in the Wnt signaling pathway to transactivate downstream target genes, such as Axis inhibition protein 2 (Axin2) [22]. Both β-catenin and TCF/LEF family members have two potential functions, acting as transcriptional activators or repressors, ultimately leading to the inhibition or activation of target genes in the Wnt signaling pathway [23].

#### TCF-1 and LEF-1 in HFSCs

TCF-1 is an initiating member of the TCF family and is induced by Notch signals during the early stages. As a pioneer transcription factor, TCF-1 is uniquely expressed in T lymphocytes in adult mammals [24]. TCF-1 plays an important role in the development of hair follicle organs and embryonic development and the self-renewal of adult tissue stem cells [25]. LEF-1 mRNA appears in the epithelium and mesenchymal compartment as early as the ectoderm stage of the placenta. In addition, LEF-1 is expressed in hair follicles, and mice in which the LEF-1 gene is knocked out show a significant reduction in hair follicles and complete coat loss [26].

#### TCF-3 and TCF-4 in HFSCs

The transcription factors TCF-3 and TCF-4 are present in mammals and the TCF-4 gene is located on the long arm of chromosome 10. As a member of the TCF/LEF transcription factor family, TCF-4 is diversified in both form and function. TCF-4 is a downstream effector of the Wnt signal transduction pathway. After Wnt signal transduction, a cascade reaction occurs, which causes β-catenin to translocate to the nucleus and interact with TCF to produce a transcriptionally active complex [27]. In the absence of Wnt protein, TCF-4 acts as a transcriptional repressor; in the presence of Wnt protein, TCF-4 acts as a transcriptional activator [28]. TCF-4 interacts with β-catenin to activate downstream target genes and promote the occurrence and development of cancer [19]. In 2014, relevant studies found a competitive relationship between TCF-3 and the Wnt/β-catenin signaling pathway [29] which has an important impact on clinical applications. As a key factor regulating the function of embryonic stem cells, TCF-3 accelerates the migration of keratinocytes to participate in skin wound healing in the microenvironment of epidermal wound repair. Additionally, it is expressed both in the entire primitive epithelium during the development of mammalian skin and in the hair follicle bulge in adult skin [30]. The hair follicle bulge is a known niche of stem cells. Cells expressing TCF-3 in hair follicle bulges are a type of self-renewing stem cell with multi-differentiation potential; they are also present in the basal layer of several other stratified epithelia and play important roles in the development of hair follicles and in determining the function of HFSCs [31,32].

### Wnt/β-catenin signaling pathway

#### Overview of the Wnt/β-catenin signaling pathway

Hair follicle regeneration depends on the activation of HFSCs during the growth cycle. Therefore, HFSCs play an important role in the development of hair follicles. HFSCs are located in the bulge below the sebaceous glands and have multiple differentiation potentials. They can differentiate and participate in the formation of hair follicles and in the maintenance of sebaceous glands and the renewal of the epidermis. The Wnt signaling pathway has a regulatory role in HFSCs, especially the classical Wnt/β-catenin pathway [33]. During hair regeneration, the stable expression of β-catenin in HFSCs at the hair germ and bulge activates the LEF/TCF complex and the transcription of downstream target genes such as c-Myc and cyclin D1, thereby promoting the activation, proliferation and directional differentiation of HFSCs [34].

The Wnt/β-catenin signaling pathway is considered the central signaling pathway for the transformation of hair follicles from the resting phase to the growth phase. It participates in all stages of the development of hair follicles and determines the differentiation fate of cells during development [35]. Many components of the Wnt signaling pathway are evolutionarily ancient and conserved, but they play an important role in cell proliferation, biological development and the regulation of normal tissue reconstruction and other life activities [36]. Its main core member is β-catenin, which plays a key role in regulating cell behavior and is necessary for maintaining hair follicles during growth. When the level of β-catenin decreases, the Wnt pathway is turned off, and when the level of β-catenin increases, the Wnt pathway is turned on [37]. Research on the Wnt/β-catenin signaling pathway has helped to promote the clinical application of HFSCs. Evidence has shown that Wnt/β-catenin contributes to the growth and development of hair follicles, which makes it a considerable prospect in the prevention and treatment of hair loss. Wnt10b/β-catenin signaling and abnormal activation of this pathway may lead to sebaceous gland tumors [18,38].

#### Mechanism of the Wnt/β-catenin signaling pathway

**Figure 1.**
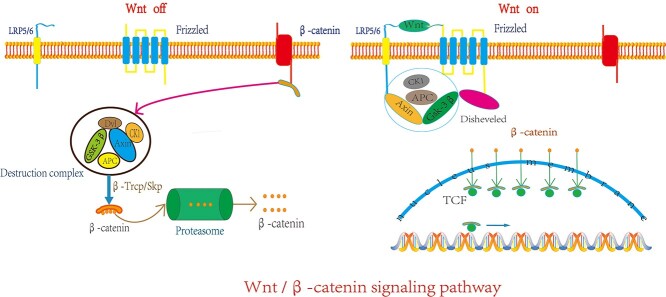
Mechanism of Wnt/β-catenin signaling pathway. When the Wnt signal is inactivated, the destruction complex ubiquitinates β-catenin to degrade it, and then it is digested in the proteasome. When the Wnt signal is activated, the Wnt protein interacts with Frizzled receptors and lipoprotein receptor-related protein 5/6 (LRP5/6). Axis inhibition protein (Axin) binds to the tail of LRP5/6, and then Disheveled (Dsh) restores the activity of β-catenin by phosphorylating and inhibiting the activity of glycogen synthase kinase-3 (GSK3) so that β-catenin accumulates and enters the nucleus with lymphoid enhancer factor/T-cell factor (LEF/TCF) transcription factors to regulate target gene transcription. *β-TrCP/SKP* β-transducin repeat-containing protein/s-phase kinase-associated protein

The genomes of most mammals, including humans, contain 19 Wnt genes, which are divided into 12 conserved Wnt subfamilies. All Wnts contain an N-terminal signal peptide for secretion, and the portion connected to the N-terminus is glycosylated [39]. The size of the Wnt protein is 40 kDa [40] and it contains many conserved cysteines which can act through paracrine or autocrine signaling. Willert *et al*. successfully purified active mouse Wnt3a for the first time using mass spectrometry in 2003, indicating that Wnts are lipid-modified [41]. The Wnt ligand is a secreted glycoprotein that is composed of a heterodimer complex. When Wnt ligand is present (pathway activation) it binds to a large complex consisting of Frizzled (FZ) and lipoprotein receptor-related protein 5/6 (LRP5/6) proteins, which causes the Disheveled (Dvl) protein to be activated after successive phosphorylation, polyubiquitination and polymerization, and the replacement of glycogen synthase kinase-3β (GSK-3β) with adenomatous polyposis coli (APC)/Axin. Following this activation, β-catenin accumulates in large quantities in the cytoplasm and enters the nucleus, where it binds to LEF-1/TCF to form a transcriptional activation complex that regulates the transcription of target genes and induces the differentiation of HFSCs [42]. When the Wnt ligand is absent (pathway inactivation), β-catenin, a complete E-cadherin and intercellular adhesion adaptor protein act as a target following the coordinated phosphorylation of creatine kinase 1 (CK1) and the APC/Axin/GSK-3β complex. The β-Trcp/Skp pathway leads to the ubiquitination and degradation of β-catenin by the proteasome (Figure 1).

#### Relationship between the Wnt/β-catenin signaling pathway and HFSCs

Some articles found, in addition, that Wnt/β-catenin signaling plays a broad role in promoting the proliferation of epidermal progenitor cells found between hair follicles during homeostasis rather than under inflammatory conditions; however, it was not necessary for maintaining the development of HFSCs [35]. During embryonic hair follicle development, the activation of the Wnt/β-catenin signaling pathway is a key initial step in the formation of hair plaques [43]. After the formation of the hair base plate, weakening of the Wnt/β-catenin signal in the upper region of the hair nail serves as a precondition for the formation of HFSCs from the hair bud to the hair cuticle; this occurs because the Wnt/β-catenin signal inhibits Sox9, and the latter is necessary for the formation of HFSCs [44]. Among the 17 Wnt proteins encoded in the mammalian genome, Wnt7 plays an important role in the Wnt/β-catenin signaling pathway, especially Wnt7b, which is not expressed in the resting phase but starts during the first growth phase after birth. If Wnt7b is knocked out after birth, the activation of HFSCs is arrested [45].

In addition, Wnt10b is also necessary for the Wnt/β-catenin signaling pathway. Lei *et al*. found that adenovirus-mediated Wnt10b protein overexpression induced the transition of hair follicles from the resting phase to the growth phase [46]. In contrast, Li *et al*. found that small interfering RNA (siRNA)-mediated gene knockout of Wnt10b can prevent hair follicles from entering the growth phase [47].

Studies have found that serine–threonine phosphate protein kinase inhibitors can promote the phosphorylation of specific Axin sites by CK1, and phosphorylated Axin is more likely to bind to the β-catenin degradation complex and promote β-catenin degradation. In addition, the Wnt target gene Axin2 is continuously expressed during the resting state of HFSCs in the carina of the outer root sheath. When β-catenin is conditionally knocked out, Axin2 expression is lost, which indicates that Axin2 expression depends on the presence of β-catenin [48]. Some studies have shown that a topical application of TPA can thicken the epidermis, which in turn makes HFSCs proliferate, leading to the activation of the Wnt signaling pathway; this accelerates the transition of hair follicles from the resting phase to the growth phase and speeds up wound healing [49].

The Wnt/β-catenin signaling pathway is considered a major regulator of epidermal stem cell renewal and HFSC lineage selection. Although previous studies have demonstrated the role of Wnt/β-catenin signaling in regulating the activity of HFSCs, understanding how to precisely stimulate HFSCs to control the transition of hair follicles from the resting phase to the growth phase through Wnt/β-catenin is an interesting topic for further exploration and clinical application.

### TGF-β/BMP signaling pathway

#### Overview of the TGF-β/BMP signaling pathway

The TGF-β family plays an important role in the development, homeostasis and repair of most tissues, including in embryonic development and bone and cartilage formation, and abnormal TGF-β signaling pathways are associated with many human diseases, such as fibrosis, immune diseases and cancer [50,51]. BMPs are the largest group of TGF-β family members [52]. Studies have found that there are >10 types of bone morphogenetic proteins in vertebrates and that these proteins have a highly conserved structure that is shared with other members of the TGF-β family [53]. BMP-2 and BMP-4 are associated with hair follicles and are currently one of the most popular research topics. Their expression also changes periodically with the hair follicle cycle [54].

The growth of hair follicles can be divided into an early growth period (I–IV) and a late growth period (V, VI). BMP-2 is not expressed during the early growth period (I–IV) and its expression gradually increases during the late growth period (V–VI), with a high expression level maintained during the early quiescent period but reduced expression during the late quiescent period [55]. BMP-4 is also periodically expressed in the inner epithelium of hair follicles, secondary hair embryos, hair papilla and adjacent extra-hair follicle dermal fibroblasts [56]. BMP-4 is highly expressed during the regressive phase of the hair follicle cycle but is expressed at low levels during the proliferative phase, which is the opposite of the expression of Wnt/β-catenin during the same period, which indicates that BMP and Wnt/β-catenin signals cooperatively regulate the balance between HFSCs and epidermal regeneration [57,58].

#### Mechanism of the TGF-β/BMP signaling pathway

**Figure 2.**
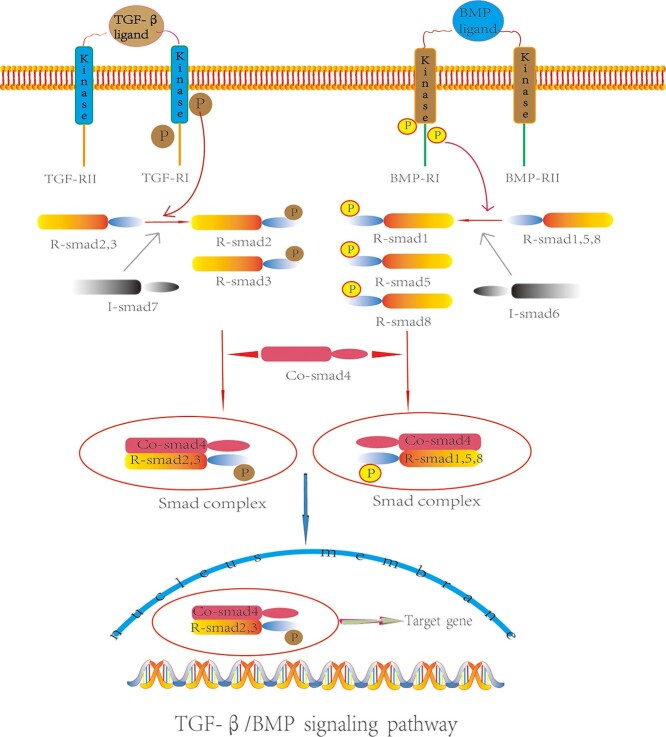
Mechanism of transforming growth factor-β/bone morphogenetic protein (TGF-β/BMP) signaling pathway. The TGF-β/BMP ligand binds to the receptor to form a binary complex, while the type II receptor activates the type I receptor, which further phosphorylates the R-Smad protein so that it can bind to Co-Smad4, enter the nucleus and activate a series of downstream target genes

Smads are a new family of signal transduction proteins with specific DNA binding ability that can enter the nucleus and produce transcription complexes to initiate the expression of downstream target genes. There are eight Smad proteins that have been confirmed by experiments and they are divided into three functional categories: R-Smads (Smad1, 2, 3, 5 and 8), Co-Smad (Smad4) and I-Smads (Smad6 and Smad7) [59]. Smads are a group of signal mediators and antagonists of the TGF-β family. Among the R-Smads, Smad2 and 3 are regulated by TGFβ, while Smad1, 5 and 8 are primarily activated by BMP. Smad4 is a key component of the typical TGFβ signaling pathway, which primarily affects the development and differentiation of hair follicles by mediating TGFβ signaling [60,61]. The TGF-β/BMP-Smad signaling pathway binds to type I receptors; BMP first phosphorylates R-Smad-1, 2, 3, 5 and 8, then binds to the Co-Smad (Smad4) to form a complex, and finally translocates to the nucleus to regulate gene transcription and inhibit cell apoptosis [62]. I-Smad6 and 7 competitively bind to type I receptors, thereby preventing the receptor-regulated phosphorylation of Smads and inhibiting TGF-β/BMP signal transduction (Figure 2). Studies have shown that BMP signaling is very active in adult hair follicle bulge stem cells [63]. When Smad1 is phosphorylated, Bmp6 levels gradually increase [64]. The BMP-MAPK signaling pathway is activated when BMP binds to type I receptors and type II receptors are re-recruited to the BMP–receptor complex [65]. Relevant studies have shown that the BMP-Smad and BMP-MAPK signaling pathways may restrict each other and jointly regulate the growth of HFSCs and the formation of bone and cartilage [66]. Abnormal expression or inactivation of the TGF-β/BMP signaling pathway can lead to a worsening of many clinical manifestations, such as the inhibition of wound healing and the aggravation of vascular malformation [67]. Through a negative feedback mechanism, the TGF-β/BMP signaling pathway determines hair follicle growth stages. However, how TGF-β/BMP signaling within the niche influences the balance between HFSC quiescence and activation is a yet-unaddressed question and requires further investigation.

#### Relationship between the TGF-β/BMP signaling pathway and HFSCs

BMP, BMP receptor and BMP inhibitors regulate cell proliferation, differentiation and apoptosis through their expression at different times and in different spaces. During the development of hair follicles, BMP-2 is expressed in the inner root sheath and participates in the development of the hair shaft. BMP-4 is expressed in the outer root sheath of the hair follicle and is an important molecule for signal transmission between epithelial cells and stromal cells. BMP-4 interacts with its inhibitor Noggin to upregulate the expression of LEF molecules, participate in bulge formation and regulate the differentiation of HFSCs into sebaceous glands, sweat glands and epidermal cells, but the specific mechanism is not clear [68]. In human bulge cells, BMP-4 also promotes upregulation of Dickkopf3 (DKK3) molecular expression [69]. DKK3 is a recently discovered secreted glycoprotein that can specifically bind to the Wnt molecule Lrp-6 to inhibit the Wnt pathway. An analysis of gene expression in bulge cells and hair follicle outer root sheath cells confirmed that compared with hair follicle outer root sheath cells and keratinocytes at the same site, human HFSCs overexpressed DKK3 [70]. BMP/DKK plays a vital role in maintaining the slow proliferation and undifferentiated characteristics of embryonic stem cells. BMP-4 is an important molecule that regulates the Wnt pathway [71]. BMP-4/DKK3 acts as a negative-feedback inhibitory signaling pathway to ensure the slow proliferation and multidirectional differentiation potential of hair follicle bulge stem cells.

The BMP signaling pathway is primarily involved in the differentiation of HFSCs. BMP ligands participate in the growth of hair follicles through the BMP signaling pathway and regulate the proliferation and differentiation of HFSCs. In recent years, BMP signaling has become a hot topic in hair follicle stem cell research [72,73]. BMP is a secreted signaling molecule belonging to the TGF-β superfamily and it exerts its biological activity through interaction with specific BMP receptors [74,75]. BMP acts as a multifunctional regulator of vertebrate development; it binds to serine and threonine receptors and phosphorylates them by interacting with other members of the growth factor family [Wnt, SHH, TGF-β, epidermal growth factor (EGF), fibroblast growth factor (FGF), Notch and neurotrophins, etc.], after which it binds to the protein Smad in the nucleus, thereby inducing the transcription of related target genes to regulate the proliferation and differentiation of HFSCs [76,77]. In the classic BMP pathway, BMP binds to the serine/threonine receptor and is phosphorylated and then translocates to the nucleus to bind to Smad1, Smad5 and Smad8 and induce the expression of downstream signaling molecules [78,79]. In a study of the BMP signaling pathway, Noggin, an antagonist of BMP, was found to be very helpful in regulating HFSCs; it can bind competitively to BMP protein receptors to antagonize the activation of this signaling pathway. Noggin is secreted and expressed by mesenchymal cells, which induce hair follicle morphogenesis in embryos. Noggin activity gradually increases during the hair follicles growth phase and weakens during the resting phase, indicating that Noggin expression changes with the hair cycle [80].

### Notch signaling pathway

#### Overview of the Notch signaling pathway

The Notch signaling pathway is a highly conserved signaling pathway that regulates the growth and development of organisms through cell–cell interactions [81] and it participates in the proliferation and differentiation of various skin cells [82]. The Notch pathway is evolutionarily ancient and plays a key role in determining cell fate and regulating the proliferation and differentiation of epidermal tissue cells during the development of mammalian embryos [83]. The Notch receptor family is a type I single transmembrane receptor protein family with four members in mice and humans. The extracellular domain of the Notch receptor contains ~36 EGF-like repetitive sequences, which are responsible for the binding of ligands. The intracellular domain of the Notch receptor contains cell division cycle 10 (CDC10)/ankyrin, a transcriptional activation domain and others.

#### Mechanism of the Notch signaling pathway

**Figure 3.**
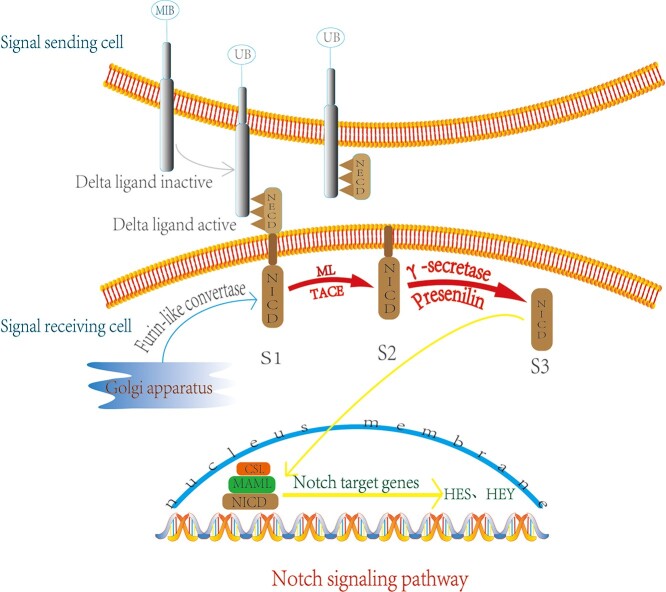
Mechanism of Notch signaling pathway. In the Golgi, the first cleavage (S1) occurs under the action of the furin-like converting enzyme. In the second lysis (S2), the extracellular region (NECD) is phagocytosed by the surface cells and then the intracellular region (NICD) is lysed for a third time under the action of γ-secretase and mutant presenilin (S3), after which it enters the nucleus, and initiates the transcription of downstream target genes. *ML* metal loprotease, *TACE* TNF-α-convertingenzyme, *CSL* CBF-1, Suppressor of hairless, Lag

In mammals, the Notch family has 4 receptors (Notch1–4), with five corresponding ligands [Delta-like 1 (Dll-1, 3, 4), Jagged-1 and Jagged-2], which bind through cell-to-cell interactions [84]. The Notch extracellular domain binds to cell ligands, and then after being hydrolyzed by the protease γ -secretase, the Notch receptor protein is cleaved three times; this releases the Notch intracellular domain (NICD), which is then transported to the nucleus where it binds to DNA, activates the transcription of target genes, such as hairy and enhancer of split (Hes), runt-related transcription factor (Runx) and Notch inhibitory membrane protein (Numb), and then activates the downstream pathways of Wnt/β-catenin signaling to exert a series of biological effects [85] (Figure 3).

#### The role of target genes in HFSCs

Hes-1 is constitutively expressed in the epidermis, eccrine glands and hair follicles and can promote cell cycle progression in the basal layer of the epidermis, thereby inducing the proliferation of keratinocytes [86]. A reduction in Hes-1 impairs differentiation and cell proliferation in the epidermis and hair follicles and reduces the pluripotency of stem cells in the skin [87]. In hair follicles, the expression of the target gene Hes-1 may be related to changes in Jagged-1 ligand gene expression [88]. Studies have found that reduced Hes-1 expression limits the ligand–receptor interaction and reduces Hes-1 signaling [89]. Numb can prevent the cleavage and nuclear translocation of the NICD, thereby blocking the activation of this signaling pathway [90]. The transcription factor Runx-1 is involved in regulating the activity of HFSCs. It is essential for the differentiation of adult HFSCs and for maintaining skin integrity. In HFSCs, Runx1 can not only promote cell proliferation but also inhibit genes such as p21 and p57. A lack of Runx1 results in the differentiation of HFSCs into sebaceous glands and a loss of regeneration function [91].

#### Relationship between the Notch signaling pathway and HFSCs

NICD is an indicator of the activation of the Notch signaling pathway. Studies have found that the Notch signaling pathway inhibitor, 4,6-diamino-2-phenylindole, can specifically block the action of γ-secretase, thereby preventing the activation of Notch [92]. The Notch signaling pathway plays an important role in maintaining the growth and development of hair follicles. It mediates the formation and re-epithelialization of hair follicles, thereby regulating the wound healing process [93].

During hair follicle morphogenesis, the Notch signaling pathway plays a small role in determining cell fate, but it is essential for the maintenance of hair follicle structure [94]. Studies have shown that Notch signaling acts on the late stages of hair follicle formation in embryos, and mice lacking Notch signaling have fine, short and curly hair [95]. In the growth phase of follicle globule embryos, three Notch receptors are expressed in partially overlapping domains: Notch1 is expressed and activated in cortical precursor cells, and Notch2 and Notch3 are expressed and activated in mitotic progeny [96]. The Notch1 intracellular domain is strongly expressed in undifferentiated hair stromal cells, hair shaft cortex and stratum corneum cells, as well as in a few cells of the outer root sheath of hair follicles and the stratum corneum of the inner root sheath [97]. However, in adult hair follicles, in the bulge area where keratinocytes and melanocyte stem cells coexist, there are very few cell subgroups with Notch1 activity [98]. Notch2 is not expressed in embryonic hair follicles but is transcribed in inner root sheath precursor cells, and Notch3 is expressed in sebaceous glands distal to Notch1 [99].

The ligand of Notch is also a type I transmembrane protein; the extracellular domain also contains multiple EGF-like repeats and the intracellular domain is very short. When Notch receptors and ligands between adjacent cells interact through their EGF-like repeat sequences, the related proteases catalyze the proteolytic cleavage of Notch receptors in the transmembrane region so that the Notch intracellular region is released from the inside of the cell membrane and enters the nucleus. The Notch intracellular region entering the nucleus can interact with the transcription factor recombination signal binding protein J (RBP-J) through the regulation of amino acid metabolism (RAM) domain to activate the transcription of the promoter containing the RBP-J recognition site, thereby regulating the expression of cell differentiation-related genes such as Hes family molecules. RAM domain-mediated Notch intracellular segment activity is related to the binding of the transcription factor RBP-J, which activates the transcription of Notch intracellular segments. In transgenic mice that specifically block the expression of RBP-J, hair follicle bulge cells differentiate into epidermal cells, inhibiting the formation of hair. The Notch/RBP-J pathway can inhibit the differentiation of HFSCs into epidermal cells and promote their differentiation into hair follicle epithelial cells [100]. The above studies indicate that the Notch signaling pathway can promote the differentiation of HFSCs into hair follicle cells, inhibit their differentiation into epidermal cells and maintain the normal differentiation of the outer root sheath, hair shaft and inner root sheath of hair follicles, as well as the normal periodic growth of hair follicles [101].

### H‌H signaling pathway

#### Overview of the HH signaling pathway

The HH pathway is an important pathway for signal transduction between the epidermis and mesenchyme and is involved in intracellular regulation. The highly conserved HH pathway is one of the main signaling pathways in the human body. It plays an important role in controlling homeostasis and tissue development. It can promote the development of the epidermis and hair follicles, repair damage and maintain the characteristics of hair follicle bulge stem cells [102]; however, when the HH pathway is abnormally activated, it can adversely affect human skin, such as in the case of basal cell carcinoma [103,104]. In vertebrates, HH proteins are divide into three types, including India hedgehog (IHH), Desert hedgehog (DHH) and sonic-hedgehog (SHH). Among them, the most in depth research has been on SHH. SHH is the most effective ligand and is widely expressed in adult tissues and hair follicles [105].

#### Mechanism of the HH signaling pathway

The HH protein is composed of an intracellular signal peptide, a highly conserved N-terminal region and a variable C-terminal domain. It can regulate the expression of N-Myc and cyclin D2 during embryonic hair follicle development and cooperate with the Wnt/β-catenin signaling pathway to participate in mature hair follicle development [106]. The classic HH signal pathway transduction process usually involves the following key components: (1) the HH ligand, (2) the cell surface receptor patched (Ptch), (3) the membrane protein smoothened (Smo), (4) the transcription factor glioma (Gli) and (5) the suppressor of fused (SuFu) protein. Ptch is a 12 transmembrane receptor protein that is composed of Ptch1 and Ptch2. It is both a receptor for HH ligands and a repressor of Smo. In the absence of HH, SuFu binds to Gli to prevent Gli from entering the nucleus and being degraded. Ptch1 is expressed in primary cilia and constitutively inhibits Smo activity. Smo is a seven-pass transmembrane G protein-coupled receptor (GPCR). It is a key effector necessary for HH signal transduction [107].

**Figure 4.**
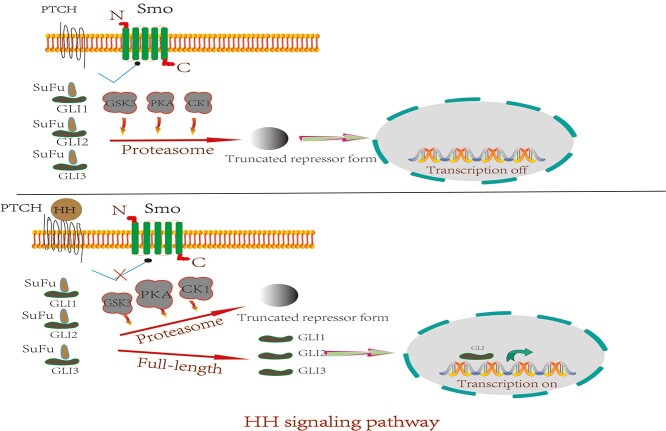
Mechanism of hedgehog (HH) signaling pathway. When there is no HH, patched (PTCH) will inhibit the activity of smoothened (Smo), leading to the termination of transcription of downstream target gene. When SHH is present, PTCH binds to SHH, releasing Smo and allowing the glioma (Gli) protein and protein kinase A (PKA) to form a macromolecular complex, such that the full-length Gli protein enters the nucleus to initiate transcription of downstream target genes

In vertebrates, Gli has three main members, Gli-1, Gli-2 and Gli-3. Relevant studies have shown that SHH-dependent Gli-1 and Gli-2 activation play a key role in the formation of hair follicles. During embryonic hair follicle development, SHH activates Gli-1 and increases the expression of Gli-1 mRNA, resulting in a faster hair growth cycle and increased hair [108]. Gli-2 is the main activator of the HH signaling pathway, Gli-3 is the main inhibitor and Gli-1 is the target of Gli-2, which together regulate signal transduction in a positive feedback manner. When the HH signal is activated, the SuFu–Gli-2 complex dissociates and Gli-2 is activated [109]. The membrane protein Smo activates the Gli transcription factor through GPCR-dependent and -independent pathways, such that the Gli transcription factor is transferred to the nucleus and activates the transcription of multiple cell signaling target genes, including HH signaling pathway components (Ptch, Gli-1) and superfamily proteins (Wnt, TGF) [110] (Figure 4). When the gene encoding the downstream transcription factor SOX9 of SHH is inactivated, epidermal repair after trauma is inhibited; HFSCs lose their niche microenvironment and can no longer migrate and proliferate to maintain the hair follicle cycle [111].

#### Relationship between the HH signaling pathway and HFSCs

Studies have shown that the SHH signaling pathway regulates the development of hair follicles by acting in the epithelium and mesenchyme, but the HH signaling pathway is not involved in the early stages of hair follicle development. In the mature stages of hair follicle development, a reduction in SHH selectively reduces the hair follicle epithelium. The proliferation of keratinocytes in the middle stages prevents epithelial cells from growing downstream [108,112]. Notably, SHH can stimulate hair follicle regeneration and accelerate hair follicle maturation by producing inductive dermis during skin wound healing in mice, indicating that SHH signaling is essential for hair follicle regeneration [113]. The absence or abnormal activation of the HH signaling pathway can lead to developmental defects and diseases, such as the development of skin basal cell carcinoma [114].

HH signaling has recently been identified as an important signaling pathway that drives the proliferative activation of HFSCs. The detailed molecular mechanisms underlying this activity necessitate further investigation.

### PI3K/AKT signaling pathway

#### Overview of the PI3K/AKT signaling pathway

As a bridge between extracellular signals and intracellular responses, the PI3K/AKT signaling pathway acts on downstream signaling molecules under the influence of a series of upstream or bypassing signaling molecules. It is an important signal transduction pathway involved in the regulation of cell growth, proliferation and differentiation. PI3K is an important kinase of inositol and phosphatidylinositol (PI). According to its different substrates, it is generally divided into three subtypes: type I, type II and type III. Among these, type I is the main type and can be divided into subclasses IA and IB. Type I is a heterodimer composed of a catalytic subunit P110 and a regulatory subunit p85. The regulatory subunit p85 consists of an amino terminus and a carboxyl terminus. The amino terminus includes one SH3 region and two proline-rich regions. The carboxyl terminus includes two SH2 domains and one region that binds to P110. As the main downstream molecule of the PI3K signaling pathway, AKT is a serine/threonine protein kinase with three subtypes, AKT1, AKT2 and AKT3, which are encoded by the PKB α, PKB β and PKB γ genes, respectively. The three AKT subtypes have >80% shared homology in their amino acid sequences, and they are very similar in structure, with common structural characteristics in the three different functional regions. Sale *et al*. used a subtype-specific antisense oligonucleotide probe strategy to study the function of AKT and its three subtypes and found that among the three AKT subtypes, primarily AKT2 was involved in the activation of downstream substrates [115].

#### Mechanism of the PI3K/AKT signaling pathway

**Figure 5.**
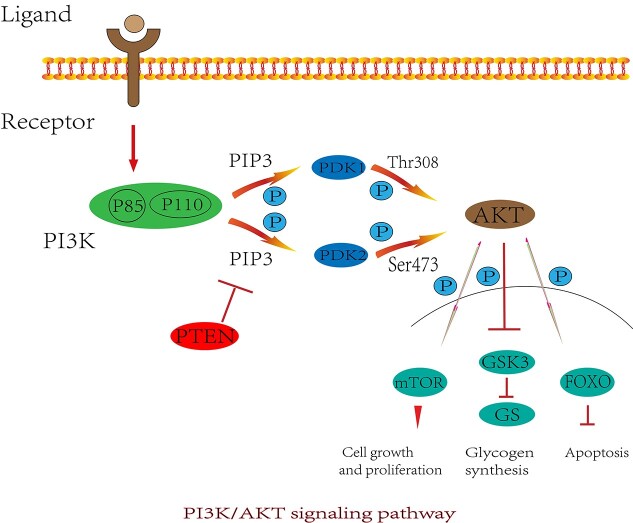
Mechanism of PI3K/AKT signaling pathway. When the ligand and receptor bind, PI3K can be activated. Phosphoinositide 3-kinase (PIP3) produced on the plasma membrane binds phosphorylated Thr308 and Ser473 through PI3K-dependent kinase 1 (PDK1) and PI3K-dependent kinase 2 (PDK2), respectively, resulting in AKT (protein kinase B) activation and action on downstream molecules

When a relevant ligand binds to the receptor, PI3K can be activated. Activation of PI3K produces the second messenger phosphatidylinositol 3,4,5-trisphosphate (PIP3) on the plasma membrane. PIP3 binds to PI3K-dependent kinase 1 (PDK1), which can phosphorylate Thr308 on the Akt protein, resulting in the activation of AKT. PI3K-dependent kinase 2 (PDK2) can phosphorylate Ser473 on AKT protein, which can also activate AKT. Activated AKT then has an important impact on downstream target proteins by activating or inhibiting related downstream molecules [such as mechanistic target of rapamycin complex (mTORC), GSK3, Forkhead box O (FOXO), etc.] [116,117]. Phosphatase and tensin homolog (PTEN) is a tumor suppressor gene. Its main function is to dephosphorylate PIP3 and convert it into phosphatidylinositol 4,5-trisphosphate (PIP2) in the cytoplasm to inhibit activation of the PI3K/AKT pathway [118] (Figure 5). To date, the PI3K/AKT signaling pathway has been shown to promote protein synthesis, affect glucose metabolism and promote fatty acid synthesis. However, the relationship between this signaling pathway and HFSCs has been less well studied.

#### Relationship between the PI3K/AKT signaling pathway and HFSCs

The PI3K/AKT signaling pathway is important for hair follicle growth and hair follicle reconstruction *in vitro*. After epidermal or HFSC injury, activated AKT phosphorylation is significantly increased and the hair follicle cell cycle is upregulated [119,120]. To further explore the effect of the PI3K/AKT signaling pathway on the hair follicle cycle, Chen *et al*. inhibited the PI3K/AKT signaling pathway with perifosine (krx-0401) and LY294002. They found that the inhibition of the PI3K/AKT signaling pathway could prevent hair follicles from transitioning from the telogen phase to the anagen phase. In addition, a PTEN inhibitor was used to enhance the activity of the PI3K/AKT signaling pathway. The PTEN inhibitor was found to induce the transformation of hair follicles from the telogen phase to the anagen phase. Relevant studies have confirmed that the PI3K/AKT signaling pathway can promote the transformation of hair follicles from telogen to early anagen, induce the proliferation of interfollicular epidermal stem cells and HFSCs, further activate the PI3K/AKT signaling pathway by releasing relevant molecules (such as cytokines and growth factors), promote hair follicle regeneration and accelerate wound healing [121–123]. Recent research results show that the interaction between long noncoding RNA5322 and miR-21 can upregulate the expression and phosphorylation of the PI3K/AKT signaling pathway, promote the proliferation and differentiation of HFSCs and play a role in skin wound healing [124,125]. In conclusion, these findings clarify that the PI3K/AKT signaling pathway affects HFSCs through different mechanisms, which provides a theoretical basis for its role in wound healing.

### Clinical applications of regulating signaling pathways in HFSCs

HFSCs represent promising candidates for stem cell-based therapies for alopecia, skin cancer, skin inflammation and skin wound healing [126]. Signaling pathways associated with the morphogenesis, development and differentiation of HFSCs provide precise targets with important functions in promoting HFSCs implicated in tissue repair and the inhibition of abnormal proliferation.

It is generally accepted that targeting the Wnt/β-catenin signaling pathway is a promising clinical treatment strategy for the regeneration of hair follicles and the downregulation of hair loss. Androgen receptor inhibitors, such as flutamide, bicalutamide, fluridil, cyproterone acetate and spironolactone, work with β-catenin to suppress Wnt signaling, thereby stimulating HFSC proliferation and differentiation and leading to hair follicle regeneration and partial hair regrowth [127–130]. Tocotrienol is a member of the vitamin E family. Topical tocotrienol-rich fraction application markedly induced anagen hair growth by upregulating the expression and nuclear translocation of β-catenin 4-fold [131,132]. Inducing and maintaining the anagen phase of the hair cycle is the basis of treating alopecia. Recent studies revealed that 3,4,5-tri-O-caffeoylquinic acid, ginkgolide B and bilobalide, morroniside and *Polygonum multiflorum* extract could promote hair follicle cycling or accelerate the onset of anagen and delay hair follicle catagen via the activation of the Wnt/β-catenin signaling pathway in HFSCs [18,133,134]. Moreover, human dermal stem/progenitor cell-derived conditioned medium enriched in Wnt3a protein promoted hair regrowth via HFSC proliferation [135]. For wound healing, the application of human alpha defensin 5 increased LGR+ stem cell migration into wound beds, resulting in bacterial presence, enhanced wound healing and hair growth through the upregulation of Wnt pathway mRNA transcripts [136].

HH pathways are known to be involved in inducing the transition from the telogen phase to the anagen phase [137]. Recent studies have reported that *Thuja orientalis*-extract and *P. multiflorum*-extract, which are traditionally used to treat baldness and hair loss, promoted hair growth by inducing the anagen phase in resting hair follicles. Additionally, early induction of β-catenin and Shh proteins in hair follicles of extract-treated patients was revealed [138,139]. Of note, multiple signaling molecules, including Gli-1, β-catenin, BMP1, BMP2 and BMP6, are regulated by a naturally occurring sesquiterpene lactone named costunolide, which stimulated the proliferation of HFSCs to improve hair growth [140]. These studies demonstrate that acting on signaling pathways related to HFSC proliferation and differentiation is an effective way to treat hair loss and other skin diseases.

Currently, the food and drug administration (FDA)-approved medications for treating baldness are Finasteride® and Minoxidil®, in addition to surgical procedures such as hair transplantation. Minoxidil has a direct effect on promoting hair growth by stimulating HFSCs and epithelial cells [141]. This compound promoted the survival of HFSCs via the activation of both the extracellular signal-regulated kinase (ERK) and protein kinase B (Akt) pathways and prevented apoptosis by increasing the Bcl-2/Bax ratio. Notably, minoxidil extends the anagen phase by activating β-catenin activity in HFSCs [142,143]. Moreover, finasteride increases the stemness of HFSCs through the AKT-dependent Wnt/β-catenin signaling pathway [144]. Although finasteride and minoxidil have been widely used, studies on how to improve their efficacy, enhance their targeting function and reduce side effects through the regulation of HFSCs require more exploration. Compared with drug treatment, platelet-rich plasma (PRP), which is made up of the growth factors released from platelets, improved hair growth and increased wound healing. After treatment with PRP, hair counts, total hair density and epidermal thickness significantly increased [126]. Human HFSCs cultured in a PRP-enhanced growth medium exhibited improved proliferation, improved Bcl-2 and FGF-7 levels, activated ERK and Akt proteins, and upregulated β-catenin [141,145].

Many drug candidates targeting HFSCs have been shown to be effective in treating alopecia, healing wounds and addressing other skin diseases. However, the mechanisms underlying hair development induced by HFSC activation are still incompletely understood, which limits the clinical application of drug candidates. Future exploration will involve the precise regulation of niche-specific HFSCs by controlling their related signaling pathways during clinical treatment.

### The networks of signaling pathways in HFSCs

**Figure 6.**
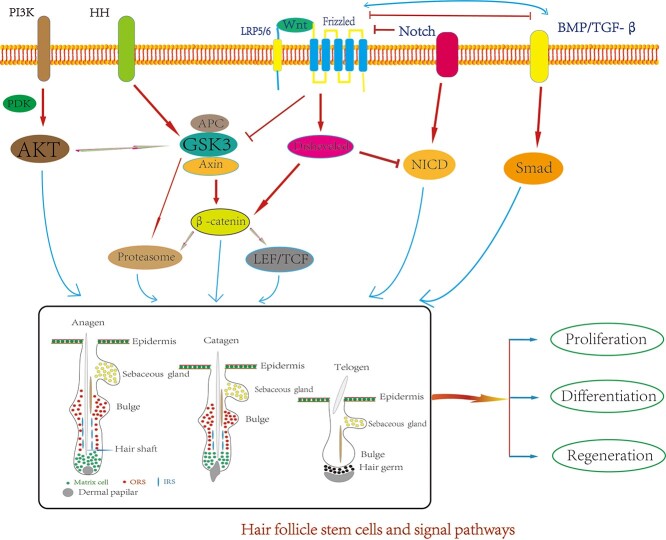
The interactive networks of signaling pathways in hair follicle stem cells. *LEF/TCF* lymphoid enhancer factor/T-cell factor, *BMP/TGF-β* bone morphogenetic protein/transforming growth factor-β, *HH* hedgehog

The Wnt pathway plays an important role during hair follicle induction, SHH participates in morphology development and advanced differentiation, Notch signaling determines the fate of HFSCs and BMP is involved in hair follicle stem cell differentiation [75]. The Wnt signaling pathway is primarily induced in HFSCs by stable β-catenin, and activation of hair follicle stem cell differentiation participates in the proliferation and regeneration of hair follicles and the epidermis. BMP and Wnt/β-catenin are two signaling pathways that coordinate to regulate the participation of HFSCs in the regeneration of the epidermis. Some members of the FGF family play an important role in hair follicle stem cell participation in the wound repair process, and the Notch1 signaling system is involved in determining whether HFSCs differentiate into hair follicles or epidermal lines. The SHH signaling pathway is required for β-catenin activity. The specific mechanisms of the signaling pathways involved in these processes have been clarified through research, and the interactions between various signaling pathways form a fine-tuned regulatory network. For example, β-catenin and TCF/LEF can act as transcription factors or repressive factors and ultimately cause the Wnt signaling pathway to be activated or inhibited. β-Catenin is the key protein of the Wnt signaling pathway, and a decrease in the level of β-catenin will turn the Wnt pathway off; in contrast, an increase in the level of β-catenin turns on the Wnt pathway [146]. BMP and Wnt/β-catenin signals regulate each other and control the balance between HFSCs and epidermal regeneration; they jointly promote the periodic growth of hair follicles and regulate normal skin tissue reconstruction [147]. The SHH signaling pathway is required for β-catenin function. The classic Wnt signaling pathway is located upstream of HH signaling. During skin development, β-catenin activation induces the expression of SHH in the epidermis [148]. The Wnt/β-catenin signaling pathway regulates the fate of HFSCs through the HH and BMP signaling pathways. In addition, the immune-related AKT/β-catenin signaling pathway plays a key role in HFSCs, promoting HF circulation and posttraumatic regeneration [149] (Figure 6). However, the specific mechanisms of action between these signaling pathways must be clarified further.

Some molecular agents have been found to modify the networks between signaling pathways in HFSCs and the hair follicle cycle. Eng is a key molecular component in the dynamic crosstalk between Wnt/β-catenin and TGF-β/BMP signaling in the hair follicle cycle. β-Catenin directly binds to the Eng promoter in a BMP signaling-dependent manner and interacts with Smad4 in a BMP/Eng-dependent context [150]. Dlx3 is a crucial transcriptional regulator of hair formation and regeneration that activates downstream of Wnt through LEF targeting and disrupts BMP signaling during hair morphogenesis [151]. In recent years, attention has been given to noncoding RNAs that participate in the progression of cellular metabolism by regulating different signaling pathways of HFSCs. lncRNA-1 and lncRNA AK015322 promoted the proliferation and differentiation of HFSCs. lncRNA-1 acts through the TGF-β1-mediated Wnt/β-catenin signaling pathway while lncRNA AK015322 targets the miR-21-mediated PI3K-AKT signaling pathway [124,152]. These molecular agents and lncRNAs provide an up-to-date view of the networks of multiple signaling pathways involved in regulating HFSCs in hair development. Nevertheless, the ways in which the intrinsic oscillation of these HFSC networks sense and influence the surrounding niche environment are still unclear. In addition, the reciprocal interactions between key niche components and key signaling pathways in HFSCs and the environment are unclear [153,154]. Therefore, future exploration is desired and will shed light on the signaling interactions between subpopulations of HFSCs and their surrounding niche environment.

### Signaling interactions with the environment of HFSCs

There are also interactions between HFSCs and other cells, such as inflammatory cells and fibroblasts. Studies have found that the BMP-MAPK signaling pathway can protect the survival of HFSCs in the inflammatory environment by enhancing the expression of sirtuin-1 [155]. In addition, during the wound healing process, molecular signaling mechanisms related to inflammation also play an important role. Wound healing begins with the inflammatory stage involving immune cells, such as neutrophils and macrophages; the process of wound healing includes homeostasis, inflammation, cell proliferation and tissue remodeling stages. Bacterial infection and continuous inflammatory reactions inhibit wound healing. HFSCs are regulated by CD4+ T cells and macrophages in the telogen phase. The hair follicle bulge can interact with recruited immune cells to overcome hair follicle telogen-phase signals [156]. Recently, polycitrate polyethyleneimine-ibuprofen reportedly showed good anti-inflammatory effects; it stimulated the differentiation of macrophages into the anti-inflammatory M2 subtype, which can accelerate hair follicle regeneration and promote wound healing [157,158].

HFSCs are promising sources for wound healing. Upon injury, HFSCs near the wound site mobilize toward it for wound bed re-epithelialization and barrier restoration. Wnt/β-catenin signaling has been shown to play a central role during wound healing, and many transcription factors take part in or are downstream of this pathway [159,160]. Wound-induced hair follicle neogenesis (WIHN) is a phenomenon that occurs in adult mammalian skin and requires Wnt signaling [161,162]. Leucine-rich repeat-containing G-protein coupled receptor 5 (LGR5) is a well-known Wnt signaling target gene and is highly expressed in HFSCs. A study showed that macrophage-derived TNF-induced AKT/β-catenin signaling in Lgr5+ HFSCs has an important function in HF cycling and WIHN after wounding [149]. Moreover, the HH pathway is important for WIHN. In scarring wounds, HH stimulates hair follicle neogenesis by establishing the dermal papilla where HFSCs reside [104]. Of note, epigenetic and Chromatin immunoprecipitation sequencing (ChIP-Seq) profiling showed that various signaling pathways are involved in hair follicle regeneration and wound repair [163]. Despite many advances, the molecular mechanisms of wound repair by HFSCs are still unclear. Much work is still needed to elucidate the crosstalk between signaling pathways in HFSCs for re-epithelialization and hair follicle neogenesis during wound healing.

### Perspective

HFSCs are one of the most common adult stem cell types in the hair follicle bulge. They are feasible for use in promoting wound healing and are currently being used in regenerative medicine. HFSCs have advantages including a wide number of sources, large numbers, minimal damage to the body during isolation, strong expansion ability *in vitro* and multidirectional differentiation potential. With the development of various technologies such as gene knockout and transgenic technology, the use of HFSCs in skin self-renewal and tissue repair and regeneration will be further developed, and our understanding and research on HFSCs will also increase. This work will play a particularly important role in tissue engineering, regenerative medicine and other fields. For example, a recent study found that adding an activated Wnt signaling pathway to dermal papilla cells can stimulate the development of hair follicles, which is a key step in hair growth. These cells can be used in wound repair and skin regeneration [164], in the treatment of androgenetic alopecia [165], in the study of skin cell carcinogenesis and as a target for gene therapy [166], all of which will provide new ideas and directions for clinical applications.

## Conclusions

In summary, hair follicles and their stem cells play an important role in promoting wound repair and maintaining normal skin function and the hair follicle cycle, during which they are regulated by LEF/TCF, Wnt/β-catenin, TGF-β/BMP, Notch, HH and PI3K/AKT signaling pathways, among others. The precise regulation of these large and complex signaling pathways effectively controls the proliferation and differentiation of HFSCs. Although the related signaling pathway network systems and specific clinical applications of HFSCs are still in the laboratory stage, current research results have indicated that there are wide range of application prospects, especially with regards to research on stem cells, and the use of tissue engineering may be valuable in solving clinical problems in the future.

## Abbreviations

AKT: Protein kinase B; APC: Adenomatous polyposis coli; APM: Arrector pili muscle; Axin: Axis inhibition protein; BMP: Bone morphogenetic protein; CK1: Creatine kinase1; DKK3: Dickkopf3; EGF: Epidermal growth factor; Gli: Glioma; GPCR: G protein-coupled receptor; GSK-3β: Glycogen synthase kinase-3β; HES: Hairy and enhancer of split; HFSCs: Hair follicle stem cells; HH: Hedgehog; LEF: Lymphoid enhancer factor; LRP5/6: Lipoprotein receptor-related protein5/6; NICD: Notch intracellular domain; PDK1: PI3K dependent kinase 1; PDK2: PI3K dependent kinase 2; PI3K: Phosphoinositide 3-kinase; PTEN: Phosphatase and tensin homolog; RBP-J: Recombination signal binding protein J; Runx: Runt-related transcription factor; SHH: Sonic hedgehog; SOX9: SRY-related high mobility group-box gene9; TCF: T-cell factor; TGF-β: Transforming growth factor-β.

## Authors’ contributions

XXW, RHY and XDC designed the experiments. XXW and YHL drafted the manuscript. XXW and RHY drew the figures and JH and JRW participated in modification of the paper. All the authors read and approved the final manuscript.

## Funding

This study was supported by the National Natural Science Foundation of China (81772136, 82172205, 81902042), the Medical Scientific Research Foundation of Guangdong Province (A2018113), the Guangdong Basic and Applied Basic Research Foundation (2021A1515011453, 2022A1515012160), the Special Fund of Foshan Summit plan (2019C002, 2019D008, 2019A006 and 2020A015) and the Foundation of Foshan City (FS0AA-KJ218–1301-0034, 2018AB003411).

## Competing interests

None declared.

## References

[1] Paus R , Cotsarelis G. The biology of hair follicles. N Engl J Med. 1999;341:491–7. 10441606 10.1056/NEJM199908123410706

[2] Stenn KS , Paus R. Controls of hair follicle cycling. Physiol Rev. 2001;81:449–94. 11152763 10.1152/physrev.2001.81.1.449

[3] Cotsarelis G , Sun TT, Lavker RM. Label-retaining cells reside in the bulge area of pilosebaceous unit: implications for follicular stem cells, hair cycle, and skin carcinogenesis. Cell. 1990;61:1329–37. 2364430 10.1016/0092-8674(90)90696-c

[4] Nicu C , Wikramanayake TC, Paus R. Clues that mitochondria are involved in the hair cycle clock: MPZL3 regulates entry into and progression of murine hair follicle cycling. Exp Dermatol. 2020;29:1243–9. 33040410 10.1111/exd.14213

[5] Geyfman M , Plikus MV, Treffeisen E, Andersen B, Paus R. Resting no more: re-defining telogen, the maintenance stage of the hair growth cycle. Biol Rev Camb Philos Soc. 2015;90:1179–96. 25410793 10.1111/brv.12151 PMC4437968

[6] Zheng Y , Du X, Wang W, Boucher M, Parimoo S, Stenn K. Organogenesis from dissociated cells: generation of mature cycling hair follicles from skin-derived cells. J Invest Dermatol. 2005;124:867–76. 15854024 10.1111/j.0022-202X.2005.23716.x

[7] Rhee H , Polak L, Fuchs E. Lhx2 maintains stem cell character in hair follicles. Science. 2006;312:1946–9. 16809539 10.1126/science.1128004 PMC2405918

[8] Vidal VP , Chaboissier MC, Lutzkendorf S, Cotsarelis G, Mill P, Hui CC, et al. Sox9 is essential for outer root sheath differentiation and the formation of the hair stem cell compartment. Curr Biol. 2005;15:1340–51. 16085486 10.1016/j.cub.2005.06.064

[9] Zhang S , Hu H, Zhang H, Liu S, Liu S, Zhang Y, et al. Hair follicle stem cells derived from single rat vibrissa via organ culture reconstitute hair follicles in vivo. Cell Transplant. 2012;21:1075–85. 22546759 10.3727/096368912X640538

[10] Oshima H , Rochat A, Kedzia C, Kobayashi K, Barrandon Y. Morphogenesis and renewal of hair follicles from adult multipotent stem cells. Cell. 2001;104:233–45. 11207364 10.1016/s0092-8674(01)00208-2

[11] Obara K , Tohgi N, Mii S, Hamada Y, Arakawa N, Aki R, et al. Hair-follicle-associated pluripotent stem cells derived from cryopreserved intact human hair follicles sustain multilineage differentiation potential. Sci Rep. 2019;9:9326. 31249324 10.1038/s41598-019-45740-9 PMC6597789

[12] Rachmin I , Lee JH, Zhang B, Sefton J, Jung I, Lee YI, et al. Stress-associated ectopic differentiation of melanocyte stem cells and ORS amelanotic melanocytes in an ex vivo human hair follicle model. Exp Dermatol. 2021;30:578–87. 33598985 10.1111/exd.14309 PMC8600567

[13] Xu ZC , Zhang Q, Li H. Human hair follicle stem cell differentiation into contractile smooth muscle cells is induced by transforming growth factor-beta1 and platelet-derived growth factor BB. Mol Med Rep. 2013;8:1715–21. 24084832 10.3892/mmr.2013.1707

[14] Li H , Ziemer M, Stojanovic I, Saksida T, Maksimovic-Ivanic D, Mijatovic S, et al. Mesenchymal stem cells from mouse hair follicles reduce hypertrophic scarring in a murine wound healing model. Stem Cell Rev Rep. 2022. 10.1007/s12015-021-10288-7. PMC9391240 35080748

[15] Shwartz Y , Gonzalez-Celeiro M, Chen CL, Pasolli HA, Sheu SH, Fan SM, et al. Cell types promoting Goosebumps form a niche to regulate hair follicle stem cells. Cell. 2020;182:578, e19–93. 32679029 10.1016/j.cell.2020.06.031 PMC7540726

[16] Fujiwara H , Ferreira M, Donati G, Marciano DK, Linton JM, Sato Y, et al. The basement membrane of hair follicle stem cells is a muscle cell niche. Cell. 2011;144:577–89. 21335239 10.1016/j.cell.2011.01.014 PMC3056115

[17] Liu G , Cheng G, Zhang Y, Gao S, Sun H, Bai L, et al. Pyridoxine regulates hair follicle development via the PI3K/Akt, Wnt and Notch signalling pathways in rex rabbits. Anim Nutr. 2021;7:1162–72. 34754958 10.1016/j.aninu.2021.09.003 PMC8556489

[18] Zhou L , Wang H, Jing J, Yu L, Wu X, Lu Z. Morroniside regulates hair growth and cycle transition via activation of the Wnt/beta-catenin signaling pathway. Sci Rep. 2018;8:13785. 30213979 10.1038/s41598-018-32138-2 PMC6137235

[19] Ravindranath A , O'Connell A, Johnston PG, El-Tanani MK. The role of LEF/TCF factors in neoplastic transformation. Curr Mol Med. 2008;8:38–50. 18289012 10.2174/156652408783565559

[20] Okumura K , Saito M, Yoshizawa Y, Ito Y, Isogai E, Araki K, et al. Pak1 maintains epidermal stem cells by regulating Langerhans cells and is required for skin carcinogenesis. Oncogene. 2020;39:4756–69. 32427988 10.1038/s41388-020-1323-3

[21] Revenco T , Lapouge G, Moers V, Brohee S, Sotiropoulou PA. Low dose radiation causes skin cancer in mice and has a differential effect on distinct epidermal stem cells. Stem Cells. 2017;35:1355–64. 28100039 10.1002/stem.2571

[22] van Noort M , Clevers H. TCF transcription factors, mediators of Wnt-signaling in development and cancer. Dev Biol. 2002;244:1–8. 11900454 10.1006/dbio.2001.0566

[23] Zhou P , Byrne C, Jacobs J, Fuchs E. Lymphoid enhancer factor 1 directs hair follicle patterning and epithelial cell fate. Genes Dev. 1995;9:700–13. 7537238 10.1101/gad.9.6.700

[24] Johnson JL , Georgakilas G, Petrovic J, Kurachi M, Cai S, Harly C, et al. Lineage-determining transcription factor TCF-1 initiates the epigenetic identity of T cells. Immunity. 2018;48:243, e10–57. 29466756 10.1016/j.immuni.2018.01.012 PMC5824646

[25] Molenaar M , van de Wetering M, Oosterwegel M, Peterson-Maduro J, Godsave S, Korinek V, et al. XTcf-3 transcription factor mediates beta-catenin-induced axis formation in Xenopus embryos. Cell. 1996;86:391–9. 8756721 10.1016/s0092-8674(00)80112-9

[26] van Genderen C , Okamura RM, Farinas I, Quo RG, Parslow TG, Bruhn L, et al. Development of several organs that require inductive epithelial-mesenchymal interactions is impaired in LEF-1-deficient mice. Genes Dev. 1994;8:2691–703. 7958926 10.1101/gad.8.22.2691

[27] Omer CA , Miller PJ, Diehl RE, Kral AM. Identification of Tcf4 residues involved in high-affinity beta-catenin binding. Biochem Biophys Res Commun. 1999;256:584–90. 10080941 10.1006/bbrc.1999.0379

[28] DasGupta R , Fuchs E. Multiple roles for activated LEF/TCF transcription complexes during hair follicle development and differentiation. Development. 1999;126:4557–68. 10498690 10.1242/dev.126.20.4557

[29] Lien WH , Polak L, Lin M, Lay K, Zheng D, Fuchs E. In vivo transcriptional governance of hair follicle stem cells by canonical Wnt regulators. Nat Cell Biol. 2014;16:179–90. 24463605 10.1038/ncb2903 PMC3984009

[30] Miao Q , Ku AT, Nishino Y, Howard JM, Rao AS, Shaver TM, et al. Tcf3 promotes cell migration and wound repair through regulation of lipocalin 2. Nat Commun. 2014;5:4088. 24909826 10.1038/ncomms5088 PMC4052366

[31] Luo W , Peterson A, Garcia BA, Coombs G, Kofahl B, Heinrich R, et al. Protein phosphatase 1 regulates assembly and function of the beta-catenin degradation complex. EMBO J. 2007;26:1511–21. 17318175 10.1038/sj.emboj.7601607 PMC1829374

[32] Howard JM , Nuguid JM, Ngole D, Nguyen H. Tcf3 expression marks both stem and progenitor cells in multiple epithelia. Development. 2014;141:3143–52. 25038042 10.1242/dev.106989 PMC4197553

[33] Myung PS , Takeo M, Ito M, Atit RP. Epithelial Wnt ligand secretion is required for adult hair follicle growth and regeneration. J Invest Dermatol. 2013;133:31–41. 22810306 10.1038/jid.2012.230 PMC3479363

[34] Rabbani P , Takeo M, Chou W, Myung P, Bosenberg M, Chin L, et al. Coordinated activation of Wnt in epithelial and melanocyte stem cells initiates pigmented hair regeneration. Cell. 2011;145:941–55. 21663796 10.1016/j.cell.2011.05.004 PMC3962257

[35] Choi YS , Zhang Y, Xu M, Yang Y, Ito M, Peng T, et al. Distinct functions for Wnt/beta-catenin in hair follicle stem cell proliferation and survival and interfollicular epidermal homeostasis. Cell Stem Cell. 2013;13:720–33. 24315444 10.1016/j.stem.2013.10.003 PMC3900235

[36] Kumar J , Swanberg M, McGuigan F, Callreus M, Gerdhem P, Akesson K. LRP4 association to bone properties and fracture and interaction with genes in the Wnt- and BMP signaling pathways. Bone. 2011;49:343–8. 21645651 10.1016/j.bone.2011.05.018

[37] Saito-Diaz K , Chen TW, Wang X, Thorne CA, Wallace HA, Page-McCaw A, et al. The way Wnt works: components and mechanism. Growth Factors. 2013;31:1–31. 23256519 10.3109/08977194.2012.752737 PMC3697919

[38] Qiu W , Lei M, Li J, Wang N, Lian X. Activated hair follicle stem cells and Wnt/beta-catenin signaling involve in pathnogenesis of sebaceous neoplasms. Int J Med Sci. 2014;11:1022–8. 25076848 10.7150/ijms.8383 PMC4115241

[39] Smolich BD , McMahon JA, McMahon AP, Papkoff J. Wnt family proteins are secreted and associated with the cell surface. Mol Biol Cell. 1993;4:1267–75. 8167409 10.1091/mbc.4.12.1267 PMC275763

[40] Zhao B , Li J, Zhang X, Dai Y, Yang N, Bao Z, et al. Exosomal miRNA-181a-5p from the cells of the hair follicle dermal papilla promotes the hair follicle growth and development via the Wnt/beta-catenin signaling pathway. Int J Biol Macromol. 2022;207:110–20. 35248611 10.1016/j.ijbiomac.2022.02.177

[41] Willert K , Brown JD, Danenberg E, Duncan AW, Weissman IL, Reya T, et al. Wnt proteins are lipid-modified and can act as stem cell growth factors. Nature. 2003;423:448–52. 12717451 10.1038/nature01611

[42] McNeill H , Woodgett JR. When pathways collide: collaboration and connivance among signalling proteins in development. Nat Rev Mol Cell Biol. 2010;11:404–13. 20461097 10.1038/nrm2902 PMC4489880

[43] Fu J , Hsu W. Epidermal Wnt controls hair follicle induction by orchestrating dynamic signaling crosstalk between the epidermis and dermis. J Invest Dermatol. 2013;133:890–8. 23190887 10.1038/jid.2012.407 PMC3594635

[44] Xu Z , Wang W, Jiang K, Yu Z, Huang H, Wang F, et al. Embryonic attenuated Wnt/beta-catenin signaling defines niche location and long-term stem cell fate in hair follicle. elife. 2015;4:e10567. 26653852 10.7554/eLife.10567 PMC4758985

[45] Kandyba E , Leung Y, Chen YB, Widelitz R, Chuong CM, Kobielak K. Competitive balance of intrabulge BMP/Wnt signaling reveals a robust gene network ruling stem cell homeostasis and cyclic activation. Proc Natl Acad Sci U S A. 2013;110:1351–6. 23292934 10.1073/pnas.1121312110 PMC3557042

[46] Lei M , Lai X, Bai X, Qiu W, Yang T, Liao X, et al. Prolonged overexpression of Wnt10b induces epidermal keratinocyte transformation through activating EGF pathway. Histochem Cell Biol. 2015;144:209–21. 25995040 10.1007/s00418-015-1330-6 PMC5572676

[47] Li YH , Zhang K, Yang K, Ye JX, Xing YZ, Guo HY, et al. Adenovirus-mediated Wnt10b overexpression induces hair follicle regeneration. J Invest Dermatol. 2013;133:42–8. 22832493 10.1038/jid.2012.235

[48] Lim X , Tan SH, Yu KL, Lim SB, Nusse R. Axin2 marks quiescent hair follicle bulge stem cells that are maintained by autocrine Wnt/beta-catenin signaling. Proc Natl Acad Sci U S A. 2016;113:E1498–505. 26903625 10.1073/pnas.1601599113 PMC4801317

[49] Trempus CS , Morris RJ, Ehinger M, Elmore A, Bortner CD, Ito M, et al. CD34 expression by hair follicle stem cells is required for skin tumor development in mice. Cancer Res. 2007;67:4173–81. 17483328 10.1158/0008-5472.CAN-06-3128 PMC2121659

[50] Carreira AC , Lojudice FH, Halcsik E, Navarro RD, Sogayar MC, Granjeiro JM. Bone morphogenetic proteins: facts, challenges, and future perspectives. J Dent Res. 2014;93:335–45. 24389809 10.1177/0022034513518561

[51] Meng XM , Nikolic-Paterson DJ, Lan HY. TGF-beta: the master regulator of fibrosis. Nat Rev Nephrol. 2016;12:325–38. 27108839 10.1038/nrneph.2016.48

[52] Chakravorty N , Hamlet S, Jaiprakash A, Crawford R, Oloyede A, Alfarsi M, et al. Pro-osteogenic topographical cues promote early activation of osteoprogenitor differentiation via enhanced TGFbeta, Wnt, and Notch signaling. Clin Oral Implants Res. 2014;25:475–86. 23600707 10.1111/clr.12178

[53] Padgett RW , Wozney JM, Gelbart WM. Human BMP sequences can confer normal dorsal-ventral patterning in the drosophila embryo. Proc Natl Acad Sci U S A. 1993;90:2905–9. 8464906 10.1073/pnas.90.7.2905 PMC46205

[54] Hansdah K , Singh N, Bouzid A, Priyadarshi S, Ray CS, Desai A, et al. Evaluation of the genetic association and mRNA expression of the COL1A1, BMP2, and BMP4 genes in the development of Otosclerosis. Genet Test Mol Biomarkers. 2020;24:343–51. 32379989 10.1089/gtmb.2019.0235

[55] Plikus MV , Mayer JA, de la Cruz D, Baker RE, Maini PK, Maxson R, et al. Cyclic dermal BMP signalling regulates stem cell activation during hair regeneration. Nature. 2008;451:340–4. 18202659 10.1038/nature06457 PMC2696201

[56] Liu Y , Zhang Y, Cui J. Recognized trophoblast-like cells conversion from human embryonic stem cells by BMP4 based on convolutional neural network. Reprod Toxicol. 2021;99:39–47. 33249234 10.1016/j.reprotox.2020.11.006

[57] Huelsken J , Vogel R, Erdmann B, Cotsarelis G, Birchmeier W. Beta-catenin controls hair follicle morphogenesis and stem cell differentiation in the skin. Cell. 2001;105:533–45. 11371349 10.1016/s0092-8674(01)00336-1

[58] Fuchs E , Tumbar T, Guasch G. Socializing with the neighbors: stem cells and their niche. Cell. 2004;116:769–78. 15035980 10.1016/s0092-8674(04)00255-7

[59] Yang L , Mao C, Teng Y, Li W, Zhang J, Cheng X, et al. Targeted disruption of Smad4 in mouse epidermis results in failure of hair follicle cycling and formation of skin tumors. Cancer Res. 2005;65:8671–8. 16204035 10.1158/0008-5472.CAN-05-0800

[60] Owens P , Han G, Li AG, Wang XJ. The role of Smads in skin development. J Invest Dermatol. 2008;128:783–90. 18337711 10.1038/sj.jid.5700969

[61] Tzavlaki K , Moustakas A. TGF-beta Signaling. Biomolecules. 2020;10:3. 10.3390/biom10030487 PMC7175140 32210029

[62] Rishikaysh P , Dev K, Diaz D, Qureshi WM, Filip S, Mokry J. Signaling involved in hair follicle morphogenesis and development. Int J Mol Sci. 2014;15:1647–70. 24451143 10.3390/ijms15011647 PMC3907891

[63] Andl T , Ahn K, Kairo A, Chu EY, Wine-Lee L, Reddy ST, et al. Epithelial Bmpr1a regulates differentiation and proliferation in postnatal hair follicles and is essential for tooth development. Development. 2004;131:2257–68. 15102710 10.1242/dev.01125

[64] Blanpain C , Lowry WE, Geoghegan A, Polak L, Fuchs E. Self-renewal, multipotency, and the existence of two cell populations within an epithelial stem cell niche. Cell. 2004;118:635–48. 15339667 10.1016/j.cell.2004.08.012

[65] Nohe A , Hassel S, Ehrlich M, Neubauer F, Sebald W, Henis YI, et al. The mode of bone morphogenetic protein (BMP) receptor oligomerization determines different BMP-2 signaling pathways. J Biol Chem. 2002;277:5330–8. 11714695 10.1074/jbc.M102750200

[66] Liu C , Goswami M, Talley J, Chesser-Martinez PL, Lou CH, Sater AK. TAK1 promotes BMP4/Smad1 signaling via inhibition of erk MAPK: a new link in the FGF/BMP regulatory network. Differentiation. 2012;83:210–9. 22387344 10.1016/j.diff.2011.12.007

[67] Cunha SI , Magnusson PU, Dejana E, Lampugnani MG. Deregulated TGF-beta/BMP Signaling in vascular malformations. Circ Res. 2017;121:981–99. 28963191 10.1161/CIRCRESAHA.117.309930

[68] Zhang W , Wang N, Zhang T, Wang M, Ge W, Wang X. Roles of melatonin in goat hair follicle stem cell proliferation and pluripotency through regulating the Wnt Signaling pathway. Front Cell Dev Biol. 2021;9:686805. 34150780 10.3389/fcell.2021.686805 PMC8212062

[69] Jamora C , DasGupta R, Kocieniewski P, Fuchs E. Links between signal transduction, transcription and adhesion in epithelial bud development. Nature. 2003;422:317–22. 12646922 10.1038/nature01458 PMC2424170

[70] Ohyama M , Kobayashi T. Isolation and characterization of stem cell-enriched human and canine hair follicle keratinocytes. Methods Mol Biol. 2012;879:389–401. 22610573 10.1007/978-1-61779-815-3_24

[71] Xiao L , Yuan X, Sharkis SJ. Activin a maintains self-renewal and regulates fibroblast growth factor, Wnt, and bone morphogenic protein pathways in human embryonic stem cells. Stem Cells. 2006;24:1476–86. 16456129 10.1634/stemcells.2005-0299

[72] Derynck R , Zhang YE. Smad-dependent and Smad-independent pathways in TGF-beta family signalling. Nature. 2003;425:577–84. 14534577 10.1038/nature02006

[73] ten Dijke P , Hill CS. New insights into TGF-beta-Smad signalling. Trends Biochem Sci. 2004;29:265–73. 15130563 10.1016/j.tibs.2004.03.008

[74] Miyazawa K , Shinozaki M, Hara T, Furuya T, Miyazono K. Two major Smad pathways in TGF-beta superfamily signalling. Genes Cells. 2002;7:1191–204. 12485160 10.1046/j.1365-2443.2002.00599.x

[75] Massague J , Chen YG. Controlling TGF-beta signaling. Genes Dev. 2000;14:627–44. 10733523

[76] Owens P , Bazzi H, Engelking E, Han G, Christiano AM, Wang XJ. Smad4-dependent desmoglein-4 expression contributes to hair follicle integrity. Dev Biol. 2008;322:156–66. 18692037 10.1016/j.ydbio.2008.07.020 PMC2642977

[77] Li AG , Koster MI, Wang XJ. Roles of TGFbeta signaling in epidermal/appendage development. Cytokine Growth Factor Rev. 2003;14:99–111. 12651222 10.1016/s1359-6101(03)00005-4

[78] Hogan BL . Bone morphogenetic proteins: multifunctional regulators of vertebrate development. Genes Dev. 1996;10:1580–94. 8682290 10.1101/gad.10.13.1580

[79] Botchkarev VA , Kishimoto J. Molecular control of epithelial-mesenchymal interactions during hair follicle cycling. J Investig Dermatol Symp Proc. 2003;8:46–55. 10.1046/j.1523-1747.2003.12171.x 12894994

[80] Zhang J , He XC, Tong WG, Johnson T, Wiedemann LM, Mishina Y, et al. Bone morphogenetic protein signaling inhibits hair follicle anagen induction by restricting epithelial stem/progenitor cell activation and expansion. Stem Cells. 2006;24:2826–39. 16960130 10.1634/stemcells.2005-0544

[81] Woodhoo A , Alonso MB, Droggiti A, Turmaine M, D'Antonio M, Parkinson DB, et al. Notch controls embryonic Schwann cell differentiation, postnatal myelination and adult plasticity. Nat Neurosci. 2009;12:839–47. 19525946 10.1038/nn.2323 PMC2782951

[82] Aubin-Houzelstein G . Notch signaling and the developing hair follicle. Adv Exp Med Biol. 2012;727:142–60. 22399345 10.1007/978-1-4614-0899-4_11

[83] Nusse R , Clevers H. Wnt/beta-catenin Signaling, disease, and emerging therapeutic modalities. Cell. 2017;169:985–99. 28575679 10.1016/j.cell.2017.05.016

[84] Artavanis-Tsakonas S , Rand MD, Lake RJ. Notch signaling: cell fate control and signal integration in development. Science. 1999;284:770–6. 10221902 10.1126/science.284.5415.770

[85] Brownell I , Guevara E, Bai CB, Loomis CA, Joyner AL. Nerve-derived sonic hedgehog defines a niche for hair follicle stem cells capable of becoming epidermal stem cells. Cell Stem Cell. 2011;8:552–65. 21549329 10.1016/j.stem.2011.02.021 PMC3089905

[86] Suen WJ , Li ST, Yang LT. Hes1 regulates anagen initiation and hair follicle regeneration through modulation of hedgehog signaling. Stem Cells. 2020;38:301–14. 31721388 10.1002/stem.3117 PMC7027765

[87] Lin HY , Kao CH, Lin KM, Kaartinen V, Yang LT. Notch signaling regulates late-stage epidermal differentiation and maintains postnatal hair cycle homeostasis. PLoS One. 2011;6:e15842. 21267458 10.1371/journal.pone.0015842 PMC3022660

[88] Dhanesh SB , Subashini C, Riya PA, Rasheed VA, James J. Pleiotropic Hes-1 concomitant with its differential activation mediates neural stem cell maintenance and radial glial propensity in developing neocortex. Cereb Cortex. 2017;27:3943–61. 27405330 10.1093/cercor/bhw207

[89] Panelos J , Massi D. Emerging role of Notch signaling in epidermal differentiation and skin cancer. Cancer Biol Ther. 2009;8:1986–93. 19783903 10.4161/cbt.8.21.9921

[90] Flores AN , McDermott N, Meunier A, Marignol L. NUMB inhibition of NOTCH signalling as a therapeutic target in prostate cancer. Nat Rev Urol. 2014;11:499–507. 25134838 10.1038/nrurol.2014.195 PMC5240474

[91] Hoi CS , Lee SE, Lu SY, McDermitt DJ, Osorio KM, Piskun CM, et al. Runx1 directly promotes proliferation of hair follicle stem cells and epithelial tumor formation in mouse skin. Mol Cell Biol. 2010;30:2518–36. 20308320 10.1128/MCB.01308-09 PMC2863705

[92] Wu X , Shen QT, Oristian DS, Lu CP, Zheng Q, Wang HW, et al. Skin stem cells orchestrate directional migration by regulating microtubule-ACF7 connections through GSK3beta. Cell. 2011;144:341–52. 21295697 10.1016/j.cell.2010.12.033 PMC3050560

[93] Shi Y , Shu B, Yang R, Xu Y, Xing B, Liu J, et al. Wnt and Notch signaling pathway involved in wound healing by targeting c-Myc and Hes1 separately. Stem Cell Res Ther. 2015;6:120. 26076648 10.1186/s13287-015-0103-4 PMC4501079

[94] Wang Z , Nan W, Si H, Wang S, Zhang H, Li G. Pantothenic acid promotes dermal papilla cell proliferation in hair follicles of American minks via inhibitor of DNA binding 3/Notch signaling pathway. Life Sci. 2020;252:117667. 32304761 10.1016/j.lfs.2020.117667

[95] Vauclair S , Nicolas M, Barrandon Y, Radtke F. Notch1 is essential for postnatal hair follicle development and homeostasis. Dev Biol. 2005;284:184–93. 15978571 10.1016/j.ydbio.2005.05.018

[96] Pan Y , Lin MH, Tian X, Cheng HT, Gridley T, Shen J, et al. Gamma-secretase functions through Notch signaling to maintain skin appendages but is not required for their patterning or initial morphogenesis. Dev Cell. 2004;7:731–43. 15525534 10.1016/j.devcel.2004.09.014

[97] Vooijs M , Ong CT, Hadland B, Huppert S, Liu Z, Korving J, et al. Mapping the consequence of Notch1 proteolysis in vivo with NIP-CRE. Development. 2007;134:535–44. 17215306 10.1242/dev.02733 PMC2583343

[98] Cai J , Lee J, Kopan R, Ma L. Genetic interplays between Msx2 and Foxn1 are required for Notch1 expression and hair shaft differentiation. Dev Biol. 2009;326:420–30. 19103190 10.1016/j.ydbio.2008.11.021 PMC2983470

[99] Massi D , Panelos J. Notch signaling and the developing skin epidermis. Adv Exp Med Biol. 2012;727:131–41. 22399344 10.1007/978-1-4614-0899-4_10

[100] Yamamoto N , Tanigaki K, Han H, Hiai H, Honjo T. Notch/RBP-J signaling regulates epidermis/hair fate determination of hair follicular stem cells. Curr Biol. 2003;13:333–8. 12593800 10.1016/s0960-9822(03)00081-2

[101] Huang C , Du Y, Nabzdyk CS, Ogawa R, Koyama T, Orgill DP, et al. Regeneration of hair and other skin appendages: a microenvironment-centric view. Wound Repair Regen. 2016;24:759–66. 27256925 10.1111/wrr.12451

[102] Sun X , Are A, Annusver K, Sivan U, Jacob T, Dalessandri T, et al. Coordinated hedgehog signaling induces new hair follicles in adult skin. elife. 2020;9:e46756. 10.7554/eLife.46756 PMC7077985 32178760

[103] Abe Y , Tanaka N. Roles of the hedgehog Signaling pathway in epidermal and hair follicle development, homeostasis, and cancer. J Dev Biol. 2017;5:12. 10.3390/jdb5040012 PMC5831796 29615568

[104] Fattahi S , Pilehchian Langroudi M, Akhavan-Niaki H. Hedgehog signaling pathway: epigenetic regulation and role in disease and cancer development. J Cell Physiol. 2018;233:5726–35. 29380372 10.1002/jcp.26506

[105] Pak E , Segal RA. Hedgehog signal transduction: key players, oncogenic drivers, and cancer therapy. Dev Cell. 2016;38:333–44. 27554855 10.1016/j.devcel.2016.07.026 PMC5017307

[106] Qian H , Cao P, Hu M, Gao S, Yan N, Gong X. Inhibition of tetrameric Patched1 by sonic hedgehog through an asymmetric paradigm. Nat Commun. 2019;10:2320. 31127104 10.1038/s41467-019-10234-9 PMC6534611

[107] Rohatgi R , Milenkovic L, Scott MP. Patched1 regulates hedgehog signaling at the primary cilium. Science. 2007;317:372–6. 17641202 10.1126/science.1139740

[108] Mill P , Mo R, Fu H, Grachtchouk M, Kim PC, Dlugosz AA, et al. Sonic hedgehog-dependent activation of Gli2 is essential for embryonic hair follicle development. Genes Dev. 2003;17:282–94. 12533516 10.1101/gad.1038103 PMC195973

[109] Tukachinsky H , Lopez LV, Salic A. A mechanism for vertebrate hedgehog signaling: recruitment to cilia and dissociation of SuFu-Gli protein complexes. J Cell Biol. 2010;191:415–28. 20956384 10.1083/jcb.201004108 PMC2958481

[110] Xin M , Ji X, De La Cruz LK, Thareja S, Wang B. Strategies to target the hedgehog signaling pathway for cancer therapy. Med Res Rev. 2018;38:870–913. 29315702 10.1002/med.21482

[111] Nowak JA , Polak L, Pasolli HA, Fuchs E. Hair follicle stem cells are specified and function in early skin morphogenesis. Cell Stem Cell. 2008;3:33–43. 18593557 10.1016/j.stem.2008.05.009 PMC2877596

[112] Motoyama J , Takabatake T, Takeshima K, Hui C. Ptch2, a second mouse patched gene is co-expressed with sonic hedgehog. Nat Genet. 1998;18:104–6. 9462734 10.1038/ng0298-104

[113] Lim CH , Sun Q, Ratti K, Lee SH, Zheng Y, Takeo M, et al. Hedgehog stimulates hair follicle neogenesis by creating inductive dermis during murine skin wound healing. Nat Commun. 2018;9:4903. 30464171 10.1038/s41467-018-07142-9 PMC6249328

[114] Salaritabar A , Berindan-Neagoe I, Darvish B, Hadjiakhoondi F, Manayi A, Devi KP, et al. Targeting hedgehog signaling pathway: paving the road for cancer therapy. Pharmacol Res. 2019;141:466–80. 30639373 10.1016/j.phrs.2019.01.014

[115] Sale EM , Hodgkinson CP, Jones NP, Sale GJ. A new strategy for studying protein kinase B and its three isoforms. Role of protein kinase B in phosphorylating glycogen synthase kinase-3, tuberin, WNK1, and ATP citrate lyase. Biochemistry. 2006;45:213–23. 16388597 10.1021/bi050287i

[116] Zhang Y , Wang X, Yang H, Liu H, Lu Y, Han L, et al. Kinase AKT controls innate immune cell development and function. Immunology. 2013;140:143–52. 23692658 10.1111/imm.12123 PMC3784161

[117] Yang J , Ikezoe T, Nishioka C, Udaka K, Yokoyama A. Bcr-Abl activates AURKA and AURKB in chronic myeloid leukemia cells via AKT signaling. Int J Cancer. 2014;134:1183–94. 23934627 10.1002/ijc.28434

[118] Lee YR , Chen M, and Pandolfi PP. The functions and regulation of the PTEN tumour suppressor: new modes and prospects. Nat Rev Mol Cell Biol, 2018; 19: 547–62. 29858604 10.1038/s41580-018-0015-0

[119] Zhang X , Zhou D, Ma T, Liu Q. Vascular endothelial growth factor protects CD200-rich and CD34-positive hair follicle stem cells against androgen-induced apoptosis through the phosphoinositide 3-kinase/Akt pathway in patients with androgenic alopecia. Dermatol Surg. 2020;46:358–68. 31478937 10.1097/DSS.0000000000002091

[120] Cheon HI , Bae S, Ahn KJ. Flavonoid Silibinin increases hair-inductive property via Akt and Wnt/beta-catenin Signaling activation in 3-dimensional-spheroid cultured human dermal papilla cells. J Microbiol Biotechnol. 2019;29:321–9. 30609881 10.4014/jmb.1810.10050

[121] Chen Y , Fan Z, Wang X, Mo M, Zeng SB, Xu RH, et al. PI3K/Akt signaling pathway is essential for de novo hair follicle regeneration. Stem Cell Res Ther. 2020;11:144. 32245516 10.1186/s13287-020-01650-6 PMC7118821

[122] Hardy KM , Yatskievych TA, Konieczka J, Bobbs AS, Antin PB. FGF signalling through RAS/MAPK and PI3K pathways regulates cell movement and gene expression in the chicken primitive streak without affecting E-cadherin expression. BMC Dev Biol. 2011;11:20. 21418646 10.1186/1471-213X-11-20 PMC3071786

[123] Koyasu S . The role of PI3K in immune cells. Nat Immunol. 2003;4:313–9. 12660731 10.1038/ni0403-313

[124] Cai B , Zheng Y, Ma S, Xing Q, Wang X, Yang B, et al. Long noncoding RNA regulates hair follicle stem cell proliferation and differentiation through PI3K/AKT signal pathway. Mol Med Rep. 2018;17:5477–83. 29393477 10.3892/mmr.2018.8546

[125] Zou ZW , Ma C, Medoro L, Chen L, Wang B, Gupta R, et al. LncRNA ANRIL is up-regulated in nasopharyngeal carcinoma and promotes the cancer progression via increasing proliferation, reprograming cell glucose metabolism and inducing side-population stem-like cancer cells. Oncotarget. 2016;7:61741–54. 27557514 10.18632/oncotarget.11437 PMC5308687

[126] Hu XM , Li ZX, Zhang DY, Yang YC, Fu SA, Zhang ZQ, et al. A systematic summary of survival and death signalling during the life of hair follicle stem cells. Stem Cell Res Ther. 2021;12:453. 34380571 10.1186/s13287-021-02527-y PMC8359037

[127] Ismail FF , Meah N, Trindade de Carvalho L, Bhoyrul B, Wall D, Sinclair R. Safety of oral bicalutamide in female pattern hair loss: a retrospective review of 316 patients. J Am Acad Dermatol. 2020;83:1478–9. 32213304 10.1016/j.jaad.2020.03.034

[128] Crabtree JS , Kilbourne EJ, Peano BJ, Chippari S, Kenney T, McNally C, et al. A mouse model of androgenetic alopecia. Endocrinology. 2010;151:2373–80. 20233794 10.1210/en.2009-1474

[129] Sovak M , Seligson AL, Kucerova R, Bienova M, Hajduch M, Bucek M. Fluridil, a rationally designed topical agent for androgenetic alopecia: first clinical experience. Dermatol Surg. 2002;28:678–85. 12174057 10.1046/j.1524-4725.2002.02017.x

[130] Sinclair R , Patel M, Dawson TL, Jr, Yazdabadi A, Yip L, Perez A, et al. Hair loss in women: medical and cosmetic approaches to increase scalp hair fullness. Br J Dermatol. 2011;165:12–8. 22171680 10.1111/j.1365-2133.2011.10630.x

[131] Beoy LA , Woei WJ, Hay YK. Effects of tocotrienol supplementation on hair growth in human volunteers. Trop Life Sci Res. 2010;21:91–9. PMC3819075 24575202

[132] Ahmed NS , Ghatak S, El Masry MS, Gnyawali SC, Roy S, Amer M, et al. Epidermal E-cadherin dependent beta-catenin pathway is phytochemical inducible and accelerates Anagen hair cycling. Mol Ther. 2017;25:2502–12. 28803863 10.1016/j.ymthe.2017.07.010 PMC5675464

[133] Zhang H , Shi Q, Nan W, Wang Y, Wang S, Yang F, et al. Ginkgolide B and bilobalide promote the growth and increase beta-catenin expression in hair follicle dermal papilla cells of American minks. Biofactors. 2019;45:950–8. 31520488 10.1002/biof.1562

[134] Bejaoui M , Villareal MO, Isoda H. Beta-catenin-mediated hair growth induction effect of 3,4,5-tri-O-caffeoylquinic acid. Aging (Albany NY). 2019;11:4216–37. 31256073 10.18632/aging.102048 PMC6628991

[135] Won CH , Jeong YM, Kang S, Koo TS, Park SH, Park KY, et al. Hair-growth-promoting effect of conditioned medium of high integrin alpha6 and low CD 71 (alpha6bri/CD71dim) positive keratinocyte cells. Int J Mol Sci. 2015;16:4379–91. 25706512 10.3390/ijms16034379 PMC4394426

[136] Lough D , Dai H, Yang M, Reichensperger J, Cox L, Harrison C, et al. Stimulation of the follicular bulge LGR5+ and LGR6+ stem cells with the gut-derived human alpha defensin 5 results in decreased bacterial presence, enhanced wound healing, and hair growth from tissues devoid of adnexal structures. Plast Reconstr Surg. 2013;132:1159–71. 24165598 10.1097/PRS.0b013e3182a48af6

[137] Oro AE , Higgins K. Hair cycle regulation of hedgehog signal reception. Dev Biol. 2003;255:238–48. 12648487 10.1016/s0012-1606(02)00042-8

[138] Zhang NN , Park DK, Park HJ. Hair growth-promoting activity of hot water extract of Thuja orientalis. BMC Complement Altern Med. 2013;13:9. 23305186 10.1186/1472-6882-13-9 PMC3637267

[139] Park HJ , Zhang N, Park DK. Topical application of Polygonum multiflorum extract induces hair growth of resting hair follicles through upregulating Shh and beta-catenin expression in C57BL/6 mice. J Ethnopharmacol. 2011;135:369–75. 21419834 10.1016/j.jep.2011.03.028

[140] Kim YE , Choi HC, Nam G, Choi BY. Costunolide promotes the proliferation of human hair follicle dermal papilla cells and induces hair growth in C57BL/6 mice. J Cosmet Dermatol. 2019;18:414–21. 29808617 10.1111/jocd.12674 PMC7379667

[141] Gentile P , Garcovich S. Advances in regenerative stem cell therapy in androgenic alopecia and hair loss: Wnt pathway, growth-factor, and mesenchymal stem cell Signaling impact analysis on cell growth and hair follicle development. Cell. 2019;8:466. 10.3390/cells8050466 PMC6562814 31100937

[142] Han JH , Kwon OS, Chung JH, Cho KH, Eun HC, Kim KH. Effect of minoxidil on proliferation and apoptosis in dermal papilla cells of human hair follicle. J Dermatol Sci. 2004;34:91–8. 15033191 10.1016/j.jdermsci.2004.01.002

[143] Kwack MH , Kang BM, Kim MK, Kim JC, Sung YK. Minoxidil activates beta-catenin pathway in human dermal papilla cells: a possible explanation for its anagen prolongation effect. J Dermatol Sci. 2011;62:154–9. 21524889 10.1016/j.jdermsci.2011.01.013

[144] Rattanachitthawat N , Pinkhien T, Opanasopit P, Ngawhirunpat T, Chanvorachote P. Finasteride enhances stem cell signals of human dermal papilla cells. In Vivo. 2019;33:1209–20. 31280211 10.21873/invivo.11592 PMC6689352

[145] Li ZJ , Choi HI, Choi DK, Sohn KC, Im M, Seo YJ, et al. Autologous platelet-rich plasma: a potential therapeutic tool for promoting hair growth. Dermatol Surg. 2012;38:1040–6. 22455565 10.1111/j.1524-4725.2012.02394.x

[146] Roose J , Clevers H. TCF transcription factors: molecular switches in carcinogenesis. Biochim Biophys Acta. 1999;1424:M23–37. 10528152 10.1016/s0304-419x(99)00026-8

[147] Silva-Vargas V , Lo Celso C, Giangreco A, Ofstad T, Prowse DM, Braun KM, et al. Beta-catenin and Hedgehog signal strength can specify number and location of hair follicles in adult epidermis without recruitment of bulge stem cells. Dev Cell. 2005;9:121–31. 15992546 10.1016/j.devcel.2005.04.013

[148] Gat U , DasGupta R, Degenstein L, Fuchs E. De novo hair follicle morphogenesis and hair tumors in mice expressing a truncated beta-catenin in skin. Cell. 1998;95:605–14. 9845363 10.1016/s0092-8674(00)81631-1

[149] Wang X , Chen H, Tian R, Zhang Y, Drutskaya MS, Wang C, et al. Macrophages induce AKT/beta-catenin-dependent Lgr5(+) stem cell activation and hair follicle regeneration through TNF. Nat Commun. 2017;8:14091. 28345588 10.1038/ncomms14091 PMC5378973

[150] Calvo-Sanchez MI , Fernandez-Martos S, Carrasco E, Moreno-Bueno G, Bernabeu C, Quintanilla M, et al. A role for the Tgf-beta/bmp co-receptor Endoglin in the molecular oscillator that regulates the hair follicle cycle. J Mol Cell Biol. 2019;11:39–52. 30239775 10.1093/jmcb/mjy051 PMC6359924

[151] Hwang J , Mehrani T, Millar SE, Morasso MI. Dlx3 is a crucial regulator of hair follicle differentiation and cycling. Development. 2008;135:3149–59. 18684741 10.1242/dev.022202 PMC2707782

[152] Si Y , Bai J, Wu J, Li Q, Mo Y, Fang R, et al. LncRNA PlncRNA1 regulates proliferation and differentiation of hair follicle stem cells through TGFbeta1mediated Wnt/betacatenin signal pathway. Mol Med Rep. 2018;17:1191–7. 29115537 10.3892/mmr.2017.7944

[153] Daszczuk P , Mazurek P, Pieczonka TD, Olczak A, Boryn LM, Kobielak K. An intrinsic oscillation of gene networks inside hair follicle stem cells: an additional layer that can modulate hair stem cell activities. Front Cell Dev Biol. 2020;8:595178. 33363148 10.3389/fcell.2020.595178 PMC7758224

[154] Chen CL , Huang WY, Wang EHC, Tai KY, Lin SJ. Functional complexity of hair follicle stem cell niche and therapeutic targeting of niche dysfunction for hair regeneration. J Biomed Sci. 2020;27:43. 32171310 10.1186/s12929-020-0624-8 PMC7073016

[155] Liu J , Xu Y, Wu Q, Ding Q, Fan W. Sirtuin1 protects hair follicle stem cells from TNFalpha-mediated inflammatory stress via activating the MAPK-ERK-Mfn2 pathway. Life Sci. 2018;212:213–24. 30292830 10.1016/j.lfs.2018.10.003

[156] Morgun EI , Vorotelyak EA. Epidermal stem cells in hair follicle cycling and skin regeneration: a view from the perspective of inflammation. Front Cell Dev Biol. 2020;8:581697. 33240882 10.3389/fcell.2020.581697 PMC7680886

[157] Okonkwo UA , DiPietro LA. Diabetes and wound angiogenesis. Int J Mol Sci. 2017;18:1419. 10.3390/ijms18071419 PMC5535911 28671607

[158] Liu W , Wang M, Cheng W, Niu W, Chen M, Luo M, et al. Bioactive antiinflammatory antibacterial hemostatic citrate-based dressing with macrophage polarization regulation for accelerating wound healing and hair follicle neogenesis. Bioact Mater. 2021;6:721–8. 33005834 10.1016/j.bioactmat.2020.09.008 PMC7516176

[159] Li B , Hu W, Ma K, Zhang C, Fu X. Are hair follicle stem cells promising candidates for wound healing? Expert Opin Biol Ther. 2019;19:119–28. 30577700 10.1080/14712598.2019.1559290

[160] Lee SA , Li KN, Tumbar T. Stem cell-intrinsic mechanisms regulating adult hair follicle homeostasis. Exp Dermatol. 2021;30:430–47. 33278851 10.1111/exd.14251 PMC8016714

[161] Wier EM , Garza LA. Through the lens of hair follicle neogenesis, a new focus on mechanisms of skin regeneration after wounding. Semin Cell Dev Biol. 2020;100:122–9. 31607627 10.1016/j.semcdb.2019.10.002 PMC7071957

[162] Ito M , Yang Z, Andl T, Cui C, Kim N, Millar SE, et al. Wnt-dependent de novo hair follicle regeneration in adult mouse skin after wounding. Nature. 2007;447:316–20. 17507982 10.1038/nature05766

[163] Adam RC , Yang H, Ge Y, Lien WH, Wang P, Zhao Y, et al. Temporal layering of Signaling effectors drives chromatin Remodeling during hair follicle stem cell lineage progression. Cell Stem Cell. 2018;22:398, e7–413. 29337183 10.1016/j.stem.2017.12.004 PMC6425486

[164] Las Heras K , Royo F, Garcia-Vallicrosa C, Igartua M, Santos-Vizcaino E, Falcon-Perez JM, et al. Extracellular vesicles from hair follicle-derived mesenchymal stromal cells: isolation, characterization and therapeutic potential for chronic wound healing. Stem Cell Res Ther. 2022;13:147. 35395929 10.1186/s13287-022-02824-0 PMC8994406

[165] Abdin R , Zhang Y, Jimenez JJ. Treatment of androgenetic alopecia using PRP to target dysregulated mechanisms and pathways. Front Med (Lausanne). 2022;9:843127. 35372424 10.3389/fmed.2022.843127 PMC8965895

[166] Chen H , Wang X, Chen Y, Han J, Kong D, Zhu M, et al. Pten loss in Lgr5(+) hair follicle stem cells promotes SCC development. Theranostics. 2019;9:8321–31. 31754399 10.7150/thno.35467 PMC6857063

